# Taxonomic revision of *Ceropegia* sect. *Huernia* (Asclepiadoideae, Apocynaceae) in Saudi Arabia with three new combinations

**DOI:** 10.3897/phytokeys.174.58867

**Published:** 2021-03-05

**Authors:** Samah A. Alharbi, Rahmah N. Al-Qthanin

**Affiliations:** 1 Biology Department, College of Applied Sciences, Umm-Al-Qura University, Makkah, Saudi Arabia Umm-Al-Qura University Makkah Saudi Arabia; 2 Biology Department, College of Sciences, King Khalid University, Abha, Saudi Arabia King Khalid University Abha Saudi Arabia

**Keywords:** Arid plants, flora of Saudi Arabia, *
Huernia
*, stapeliads, stem-succulent

## Abstract

This study provides a taxonomic revision for Ceropegia sect. Huernia in the flora of Saudi Arabia. Forty-six quantitative and qualitative morphological characters were analysed using principal component analysis (PCA), principal coordinates analysis (PCoA) and the unweighted pairs group using mean average (UPGMA) to separate and help delimit taxa. We propose to reduce the number of species reported in Saudi Arabia from 11 to four: *C. khalidbinsultanii***comb. nov.**, *C. laevis*, *C. lodarensis* and *C. macrocarpa*. This study also suggested reducing two names to varietal level under *C. lodarensis* (var. foetida**comb. nov.** and var. rubrosticta**comb. nov.**). A key to the species, detailed morphological descriptions, illustrations, distribution maps, ecology, etymology and preliminary conservation assessments are provided that follow IUCN criteria.

## Introduction

The stapeliads, essentially stem-succulent leafless members of the tribe Ceropegieae (Asclepiadoideae, Apocynaceae), comprise 357 species placed in 31 genera. All of these were reduced to sections of *Ceropegia* after a broad circumscription of the genus, based on a recent molecular study (Bruyns et al. 2017). The stapeliads are widely distributed in semi-arid to arid areas of the Old World from southern Africa north-eastwards to India and Myanmar (Bruyns 2005).Their flowers are amongst the most beautiful of the dicotyledons, as well as amongst the most complex with almost all of them scented of carrion or bad fish and similar pungent odours (Pillans 1920; Court 2000). They exhibit an extraordinarily wide range of floral shapes and sizes and a wide range of complicated structures in the centre of the flower that are associated with the pollination process (Bruyns 2005). The flowers are specialised exclusively for fly pollination and this diversity appears to have arisen in response to the wide range of sizes of flies that are present in the region, combined with the wide spectrum of geological and topographical niches in the area (Bruyns 2005).

Ceropegia sect. Huernia (R.Br.) Bruyns (formerly the genus *Huernia* R.Br.) has the widest distribution of all stapeliads, extending from west of Al-Madinah in Saudi Arabia, north of the Tropic of Cancer, to near Cape Town in South Africa, to the south of the Tropic of Capricorn (Plowes and McCoy 2003). As can be expected in a section with a range this large, *Huernia* has a great number of species and subspecies, with over 54 species currently recognised, making it the most diverse section in the stapeliads (Bruyns et al. 2017). It is distinguished from other angled-stemmed stapeliads by the leaf-rudiments without stipular denticles; corona very rarely raised above the base of the tube on a stipe, outer series spreading at the base of the tube and often partially fused to it, tube often with an annular thickening around the mouth, but not entirely formed by an annulus (Bruyns 2014). Plants of *Huernia* have almost identical stems, so that the species cannot always be identified accurately without flowers. Flowers also sometimes show a variety of forms within a single species, such as *H. humilis* and *H. thuretii* from South Africa (Bruyns 2005). Consequently, the number of species approved for *Huernia* has varied widely over time: 45 for White and Sloane (1937), 64 for Leach (1988), 49 for Bruyns (2005) and a little over 54 currently.

In Saudi Arabia, sect. Huernia is restricted to the mountainous area of the western and south-western part of the country (the mountains of Sarat and Hejaz) (Collenette 2000). The famous plant collector, Mrs I. S. Collenette, was the first to collect *Huernia* from Saudi Arabia. Between 1972 and 1998, she collected several apparently undescribed species of *Huernia* with relatively large papillate flowers (Plowes 2014). These specimens have been deposited at the Royal Botanic Gardens herbaria at Kew (K) and Edinburgh (E). Only one species had previously been described by Field, in 1980, in the course of naming her collections at Kew; this was *H. saudi-arabica* D.V.Field (Field 1980). Somewhat later, in 1985, Collenette published her first book on Saudi Arabian plants, *An Illustrated Guide to the Flowers of Saudi Arabia*. She recorded two species, *H. lodarensis* Lavranos and *H. saudi-arabica* and four unnamed species (Collenette 1985). In her subsequent publications (Collenette 1998, 1999, 2000), three species were recognised: *H. arabica* N.E.Br., *H. laevis* J.R.I.Wood and *H. saudi-arabica*. Some of the five species which lacked names have an affinity to *H. boleana* M.G.Gilbert and *H. lodarensis*. Soon after, Al-Hemaid published the name *H. haddaica* for the specimen *Collenette 5944* from Al-Hadda (Al-Hemaid 2001), but this name was not validly published (Goyder and Al-Hemaid 2009). Tom A. McCoy collected a similar plant in 1999 from Khamis Mushait, which was described in 2003 as *H. khalidbinsultanii* Plowes & McCoy (Plowes and McCoy 2003). Just two years later, in 2005, Bruyns reduced the number of *Huernia* species in north-eastern Africa and Arabia to 14 species. In his treatment, he considered *H. saudi-arabica* and *H. khalidbinsultanii* to be synonyms of *H. lodarensis* and *H. arabica* to be a synonym of *H. penzigii* N.E.Br. (Bruyns 2005). However, this was not accepted by Plowes who published seven new names for Collenette’s other Saudi Arabian collections of *Huernia* that lacked names (Plowes 2012; Plowes 2014). Thus, from the taxonomic perspective of Plowes, *Huernia* in Saudi Arabia was represented by 11 species *H. anagaynensis* Plowes, *H. arabica*, *H. asirensis* Plowes, *H. collenetteae* Plowes, *H. decaloba* Plowes, *H. foetida* Plowes, *H. khalidbinsultanii*, *H. laevis*, *H. radhwana* Plowes, *H. rubrosticta* Plowes and *H. saudi-arabica*.

Plowes’ classification of Saudi Arabian *Huernia* needs further investigation. His taxonomic treatment of several taxa was based on a single photo (e.g. *H. decaloba*) or a single specimen (e.g. *H. anagaynensis*, *H. radhwana*, *H. foetida*, *H. khalidbinsultanii*). Furthermore, morphological characters used by Plowes are not strong enough for delimiting species within sect. Huernia. For example, corolla tube size and its exterior colour, the number of flowers in the inflorescence and flower odour were used as diagnostic characters to separate *H. anagaynensis*, *H. radhwana* and *H. asirensis*. Observations of the first author have shown that such characters are not constant in this complex group. In addition, Plowes’ description of the species is not sufficiently detailed and is not even clearly enough illustrated for one to distinguish between these closely-related species. Moreover, it is unclear how the Saudi Huernias are distinguished from closely-allied species, such as *H. boleana* and *H. lodarensis*. There is, therefore, a need for much more sampling and detailed examination before a conclusive taxonomic statement on Saudi Arabian *Huernia* can be made.

Remarkably, sect. Huernia has received little taxonomic attention in Saudi Arabia, other than Plowes’ work. Taxonomic revision of this plant group in Saudi Arabia is urgently needed. Plants of sect. Huernia are commonly used for diabetes treatments in traditional medicine in the western and south-western regions of Saudi Arabia (Hamam et al. 2018). Ongoing investigations on the medicinal value of Saudi Huernias have been performed (Ali et al. 1984; Mossa and Abdul Hameed 1991; Almehdar et al. 2012; Alzahrani et al. 2015; El Sayed et al. 2018, 2020; Hamam et al. 2018). However, in some recent studies (e.g. Alzahrani et al. 2015; Hamam et al. 2018), the species of sect. Huernia investigated was identified as Huernia sp. nov. aff. boleana according to Collenette (1999). The precision and usefulness of medicinal investigations on sect. Huernia of Saudi Arabia will be increased by a detailed taxonomic treatment. It will also enable studies on their conservation status to be made, as it would appear that some of them are being severely threatened by overgrazing, infrastructure and housing development (Abulfatih and Nasher 1988; Collenette 2000; Plowes 2012).

The objectives of the present study are: 1) to revise Ceropegia sect. Huernia in the flora of Saudi Arabia, 2) to examine morphological characters in detail and try to find new ones that can be used in the classification of the section in Saudi Arabia, 3) to investigate the relationship between Saudi Arabian *Huernia* and other allied species in the Arabian Peninsula and 4) to provide a diagnostic key for the species in Saudi Arabia. This will enable us to test whether the taxonomic treatment in Plowes (2012) was justified.

## Material and methods

### Taxon sampling

Twenty individuals of *H. asirensis* and *H. collenetteae* were sampled from the Ash Shafa area in Al-Taif Province, western Saudi Arabia (21°3.6583'N, 40°20.1917'E) during several expeditions to the area between September 2010 and May 2011. Specimens were preserved for each collection in a mixture of Formalin, Glycerol and Water (in the ration 2:1:20). Herbarium specimens were then made from this preserved material as described in Leach (1995). Voucher specimens were deposited in the herbarium of Umm Al-Qura University (UQU, proposed abbreviation). Pickled and dried specimens of *Huernia* from Saudi Arabia and the Arabian Peninsula, generally, were examined at the herbaria at Kew (K) and Edinburgh (E).

### Morphological characteristics

The morphological characters were examined and recorded from the available specimens (one *H. anagaynensis*, 12 *H. asirensis*, 10 *H. collenetteae*, one *H. foetida*, one *H. laevis*, one *H. radhwana*, one *H. rubrosticta* and one *H. saudi-arabica*). Since some characters are difficult to interpret in dry specimens, dried specimens of *H. collenetteae*, *H. rubrosticta* and *H. saudi-arabica* are excluded from the morphometric analysis. For species where material was unavailable, such as *H. arabica* and *H. khalidbinsultanii* and the closely-related species from Ethiopia and the Arabian Peninsula *H. boleana* and *H. lodarensis*, measurements and character-states have been extracted from the relevant literature (Gilbert 1975; Leach 1976; Field 1980; Albers and Meve 2002; Plowes and McCoy 2003; Plowes 2012; Plowes 2014). Height of the plant, odour and colour of flowers were immediately documented in the field. Floral characters were examined using a NOVEX AP-8 binuclear microscope. Pollinia, inner corona and apices of papillae were examined using a XSZ-107BN compound optical microscope. Quantitative morphological characteristics were measured using a ruler; Suppl. material 1: Appendix 1 illustrates how the plant parts were measured. Initially, 69 characters were recorded, but 23 proved invariant leaving 46 (19 quantitative and 27 qualitative) for the analysis (Table 1, 2). The data were entered into an Excel spreadsheet and were later transformed into a format suitable for morphometric analysis. These morphological characteristics were used as the basis for our taxonomic revision for the species of sect. Huernia in Saudi Arabia. The features are richly illustrated using ibisPaint X ver.6.4.3 for Android, which allows visual comparison of the species.

**Table 1.** Nineteen quantitative morphological characters used in morphometric analysis of Ceropegia sect. Huernia in Saudi Arabia. All measured in mm.

No.	Character	No.	Character
1	length of branches	11	diam. of corolla tube at mouth
2	length of tubercles on branches	12	length of papillae in throat of corolla (max.)
3	width of base of tubercles	13	thickness of papillae at base
4	number of flowers per inflorescence	14	length of intermediate lobes
5	length of pedicel	15	length of corolla lobe
6	diam. of pedicel	16	width of corolla lobe at base
7	length of sepals	17	diam. of outer corona
8	width at base of sepals	18	length of inner corona
9	diam. of corolla	19	width of Inner corona at base
10	length of corolla tube		

**Table 2.** Twenty-seven qualitative morphological characters and character states used in morphometric analysis of Ceropegia sect. Huernia in Saudi Arabia.

No.	Characters	Character state
1	stem grooves between tubercle rows	1. deep
		2. shallow
2	flower smell	1. no bad smell
		2. faint or no bad smell
		3. very foetid
3	flowers opening	1. successively
		2. simultaneously
4	pedicel tapering towards the point of flower attachment	1. not tapering
		2. slightly tapering
		3. conspicuously tapering
5	pedicel growth direction	1. spreading and holding flower facing horizontally
		2. ascending holding flower facing upwards
6	corolla shape	1. shallow bowl shape
		2. broadly funnel-shaped, margin weakly bulging like an annulus
		3. tubular-campanulate
		4. campanulate
7	corolla lobe apex groove	1. absent
		2. present, but not deep (concave)
		3. present and deep (channel)
8	corolla inside surface texture	1. tube base smooth, tube throat and lobes papillate
		2. glabrous with very short papillae at apices of lobes
9	corolla tube	1. cylindrical
		2. pentagonal
10	shape of papillae inside corolla	1. very small, wart-like
		2. slender (hair-like)
		3. conical, compressed
		4. cylindrical or slightly compressed
11	corolla inside (background colour)	1. cream
		2. shiny creamy-yellow
		3. white
12	corolla inside (colour pattern)	1. purple
		2. brownish-red (maroon)
13	corolla exterior (colour pattern)	1. dark spots especially on the lower portion of corolla tube
		2. pale spots uniformly scattered
		3. dark spots on the upper half
		4. no spots
14	corolla tube interior (colour pattern)	1. uniform colour (purplish-red)
		2. shiny irregular broad streaks
		3. concentric broken lines and dashes
		4. concentric short dashes
		5. uniform colour (cream)
		6. dots
15	corolla lobes colour	1. uniform colour (purplish-red)
		2. shiny irregular broad streaks
		3. irregular shaped fine short lines and dashes
		4. dots
		5. deep coloured areas concentrated between the lobes; apex is streaked with irregular short lines and dashes
		6. irregular shaped short lines and dashes
16	corolla lobes spreading	1. ascending
		2. reflexed
		3. slightly spreading
		4. spreading with recurved apices
17	corolla lobe shape	1. deltoid-acute
		2. deltoid-acuminate
		3. deltoid-caudate
18	outer corona colour	1. blackish-maroon
		2. cream at the base of the lobes then gradually turning maroon towards blackish-maroon apex
19	outer corona shape	1. discrete, with 5 lobes
		2. disc
20	outer corona lobe shape	1. subquadrate
		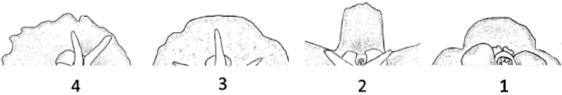
		2. rectangular
		3. short and broad rounded lobes
		4. no distinct lobes
21	outer corona lobe apex	1. shallowly bifid
		2. slightly crenate
		3. crenate
		4. emarginate
		5. slightly emarginate
		6. mucronate
		7. bifid
		8. dentate
22	outer corona fleshy tubercle	1. present
		2. absent
23	inner corona shape	1. exceeding anthers and meeting in centre
		2. shorter than anthers
		3. adpressed to anthers in their lower half then rising up connivent and then diverging towards apices
24	inner corona dorsal gibbosity	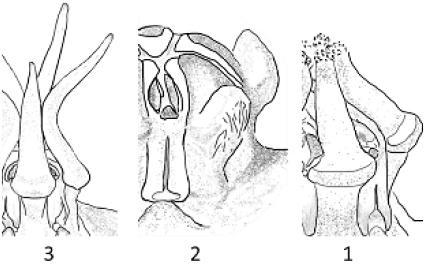
		1. broadened transversely and conspicuously gibbous
		2. ascending obtuse and conspicuously gibbous
		3. inflated transversely not conspicuously gibbous
25	inner corona apices	1. obtuse
		2. acute
		3. taper to fine, slender points
26	inner corona colour	1. maroon
		2. purple with cream at base
		3. ivory white with a few maroon spots at the tips
		4. ivory white
		5. purplish-black with cream at base
27	inner corona apex texture	1. bristly
		2. smooth
		3. minutely scabrous

### Data analysis

Qualitative characters were coded as multi-state, for example (1. cream, 2. shiny creamy-yellow, 3. white). Quantitative variables were standardised using the R studio version (2017) scale balance function to remove bias due to size alone, following Katapally and Muhajarine (2014). The standardised data were analysed with R studio package Factor Analysis of Mixed Data (FAMD) version 1.2.3; this method included principal component analysis (PCA), used here to extract relevant information from high-dimensional datasets. Cluster analysis including principle coordinates analysis (PCoA) and unweighted pairs group using mean average (UPGMA) were carried out using the statistical software Minitab ver.18.1.1.0 (Minitab, Inc., State College, PA).

### IUCN Preliminary Conservation Status

To assess the conservation status of each taxon, the guidelines for the IUCN Red List Categories and Criteria version 13 (IUCN Standards and Petitions Subcommittee 2017) and the guidelines for the Application of the IUCN Red List Criteria at the regional and national levels version 4.0 (IUCN 2012) were followed. Current threats and point distribution data were gathered from field observations and from the available scientific literature. These distributional data were then input into the GeoCAT software (Bachman et al. 2011), which, in turn, calculated two main spatial metrics: the Extent of Occurrence (EOO) and Area of Occupancy (AOO). If the EOO were less than the AOO, the EOO was set equal to the AOO to ensure consistency with the definition of the AOO as an area within the EOO following the IUCN guidelines (IUCN Standards and Petitions Subcommittee 2017). Criterion B was only used for the species assessment due to data availability. Distribution maps were created using ArcGIS Online (Esri, ‘Topography’).

### Data resources

The data underpinning the analyses reported in this paper are deposited at GBIF, the Global Biodiversity Information Facility: https://doi.org/10.15468/6n2rgz.

## Results

**Figure 1.**
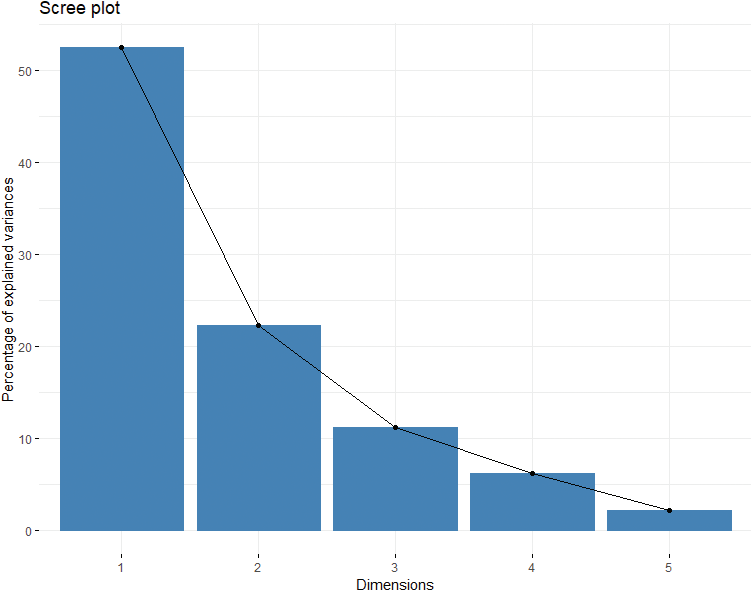
The proportion of variance retained by the different dimensions (axes), in PCA.

The first two axes of PCA accounted for 74.7% of the overall variation (Fig. 1). Screen plot Eigenvalues for identification of principal components and Boxplot showing differences in morphological characters can be seen in the supplementary information Suppl. material 1: Appendices 2 and 3, respectively.

**Figure 2.**
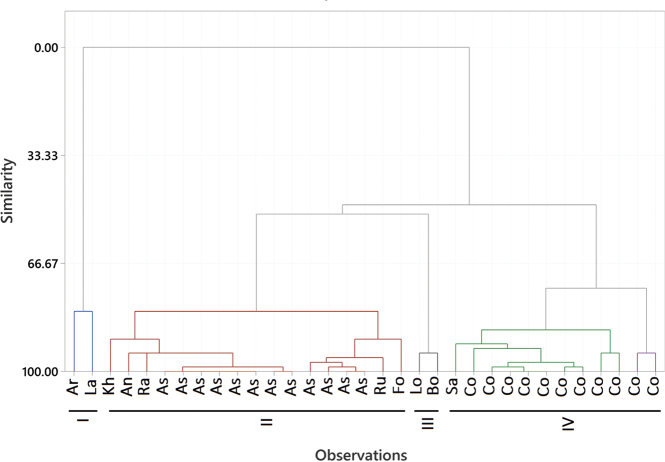
Unweighted pair-group method with arithmetic mean (UPGMA) phenogram resulting from cluster analysis. Explanations: Ar-*Huernia arabica*, La- *H. laevis*, Kh- *H. khalidbinsultanii*, Ra- *H. radhwana*, An- *H. anagaynensis*, As- *H. asirensis*, Ru- *H. rubrosticta*, Fo- *H. foetida*, Lo- *H. lodarensis*, Bo- *H. boleana*, Sa- *H. saudi-arabica*, Co- *H. collenetteae*.

Cluster analysis by UPGMA of quantitative and qualitative data indicated the presence of four clearly-distinguished groups: Group 1, with *H. arabica* and *H. laevis* is the furthest away from all other groups; Group 2, consisting of *H. asirensis*, *H. anagaynensis*, *H. khalidbinsultanii*, *H. radhwana*, *H. foetida* and *H. rubrosticta*; Group 3, with *H. lodarensis* and *H. boleana*; and Group 4, with *H. collenetteae* and *H. saudi-arabica* (Fig. 2).

**Figure 3.**
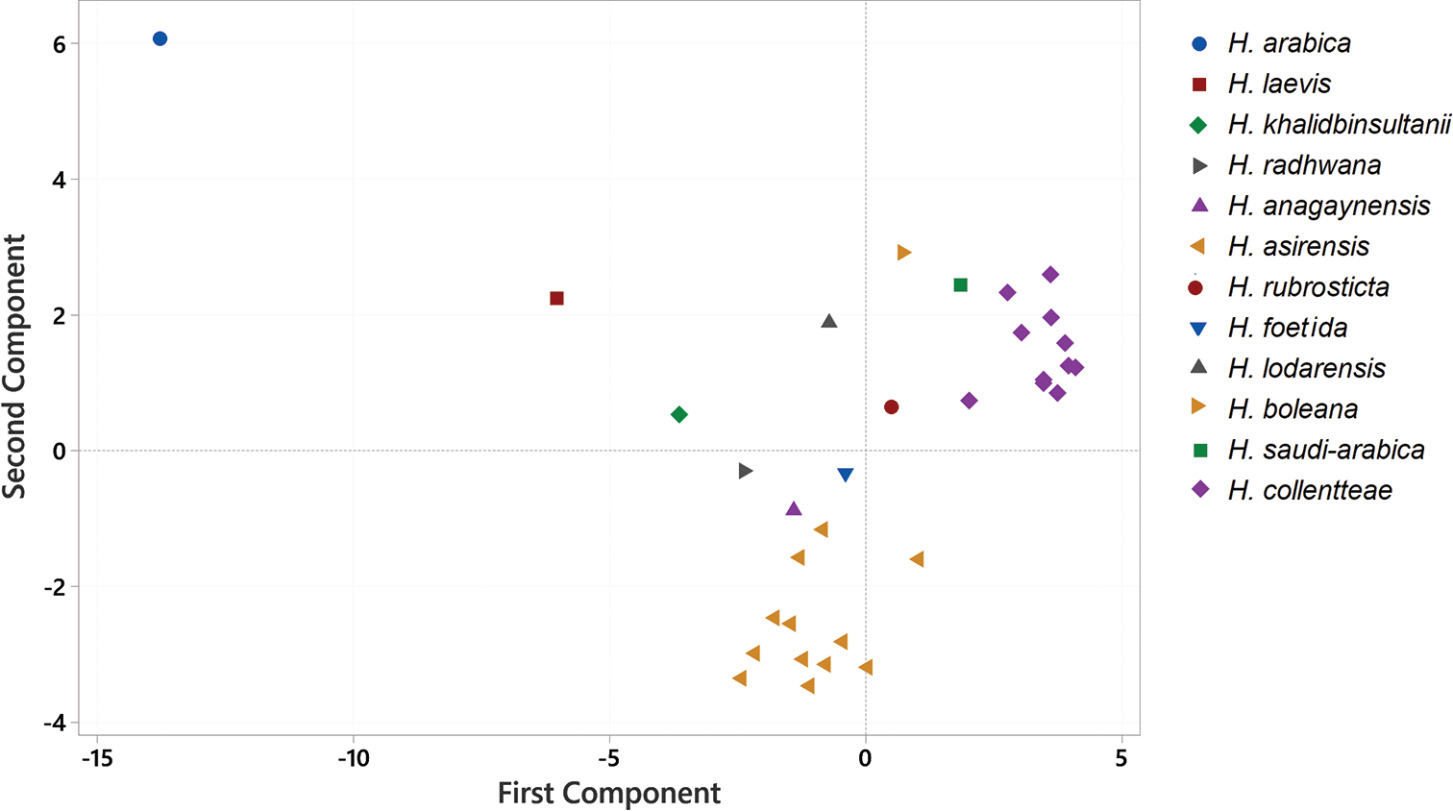
PCoA representation of morphological data of accessions of sect. Huernia. Principal Component axis 1 and 2.

PCoA separated 33 specimens into four distinct groups, corresponding largely to those obtained by UPGMA. Group 1 and 2 represent *H. arabica* and *H. laevis*, respectively, which were well-separated from the others. Accessions of *H. asirensis* were clustered together in one group and weakly separated from *H. anagaynensis*, *H. radhwana*, *H. foetida* and *H. khalidbinsultanii*. Accessions of *Huernia collenetteae* and *H. saudi-arabica* were grouped in one cluster in the positive axes and weakly separated from individuals of *H. rubrosticta*, *H. lodarensis* and *H. boleana* (Fig. 3).

The most important characters, contributing to the separation of the groups, were corolla characteristics (corolla shape, corolla tube diam. and shape, corolla lobes spreading, corolla colour patterns and papillae shape) and corona characteristics (outer corona shape, diam. and lobe shape; inner corona length, apex and dorsal gibbosity) Table 3.

**Table 3.** Eigenvalues in two principal components (PC1 and PC2) of value relative to 46 morphological characters of sect. Huernia in Saudi Arabia.

Characters	PC1	PC2
width of base of tubercles (mm)	0.078	-0.038
length of branches (mm)	0.003	-0.107
length of tubercles on branches (mm)	0.050	0.254
stem grooves between tubercle rows	0.132	-0.208
flower smell	-0.079	-0.317
number of flowers per inflorescence	0.014	-0.023
flowers opening	-0.025	-0.261
length of pedicel (mm)	0.171	0.053
diam. of pedicel (mm)	0.162	0.019
pedicel tapering toward the point of flower attachment	0.139	-0.077
pedicel growth direction	0.194	0.268
length of sepals (mm)	0.141	-0.202
width at base of sepals (mm)	0.125	-0.043
corolla shape	-0.252	0.009
corolla lobe apex groove	0.036	0.027
corolla inside surface texture	-0.085	0.072
corolla tube	0.160	-0.215
shape of papillae inside corolla	0.241	0.092
thickness of papillae at base	0.160	0.074
corolla inside (background colour)	0.171	0.142
corolla inside (colour pattern)	-0.108	0.011
corolla exterior (colour pattern)	0.076	-0.224
corolla tube interior (colour pattern)	0.158	0.153
corolla lobes colour	0.075	-0.158
corolla lobes spreading	0.172	0.011
width of base of tubercles (mm)	0.128	0.229
corolla lobe shape	0.194	0.110
diam. of corolla	0.089	0.022
length of corolla tube (mm)	0.198	-0.139
diam. of corolla tube at mouth (mm)	0.209	-0.045
length of intermediate lobe (mm)	0.156	0.004
length of corolla lobe (mm)	0.186	-0.085
width of corolla lobe at base (mm)	0.131	-0.077
outer corona colour	0.043	0.056
outer corona shape	-0.031	-0.237
outer corona lobe shape	0.091	-0.259
outer corona lobe apex	0.164	0.058
outer corona fleshy tubercle	-0.039	-0.075
diam. of outer corona (mm)	0.216	0.080
inner corona shape	0.219	-0.183
length of inner corona (mm)	0.244	0.054
width of inner corona at base (mm)	0.173	-0.029
inner corona dorsal gibbosity	0.255	0.071
inner corona base end	0.214	-0.210
inner corona apices	0.031	-0.265
inner corona apex texture	0.100	-0.072

## Discussion

In the experience of the present authors, sect. Huernia is a difficult group in the flora of Saudi Arabia and it has not received adequate attention. Perhaps the most comprehensive account is Plowes (2012). However, the diagnostic characters that were used by Plowes are questionable. In this study, multivariate analysis of 46 quantitative and qualitative morphological characters was conducted. Analyses by PCA, PCoA and UPGMA were used to determine the characters that were useful in the taxonomy of species of sect. Huernia in Saudi Arabia. Vegetative characters, such as habit, the number of angles into which the tubercles are arranged along the branches and leaf-rudiments are extremely variable across sec. Huernia, especially amongst the southern African species (Bruyns 2005). However, these characters were valueless in the taxonomy of Arabian members, due to their considerable similarity between species. Therefore, the species were differentiated mainly on the basis of their floral characters.

In the multivariate analysis, accessions of *H. radhwana* (Fig. 6D), *H. asirensis* (Fig. 6F), *H. anagaynensis* (Fig. 6G) and *H. khalidbinsultanii* (Fig. 6H) grouped into one large cluster (Figs 2, 3). Plowes (2012) distinguished between those species by: 1) size of the corolla tube, 2) colour of the exterior of the corolla, 3) the number of flowers per inflorescence and the succession of their opening and 4) the odour of the flowers. A careful examination of the type specimens suggests that they are all samples of a single species and these characters have all proved to be unreliable in our experience. The first point is not of any value since the size of the corolla tube in *H. radhwana*, *H. anagaynensis* and *H. khalidbinsultanii* is easily accommodated within the known range of *H. asirensis*. Variation found in specimens of sect. Huernia from Wadi Thee Gazal has also demonstrated the invalidity of the second point. In the case of the third, Plowes distinguished *H. radhwana* from other species in the group by its solitary flowers. This is encountered often in *H. asirensis*, where several specimens were found to have few flowers (2–4) that opened in succession. In respect of the fourth point, we do not consider that the odour of the flower is a prominent character. We observed that the foetid odour in flowers of *H. asirensis* becomes faint or vanishes completely after all pollinia were removed. In addition, this character is not considered diagnostic, as it is impossible to observe in preserved specimens. Thus, our results paper suggest that Plowes’ names for these species with slender papillae and tubular-campanulate flowers should all be included as synonyms under *H. khalidbinsultanii*, since it is the first valid name from this group.

In 2005, Bruyns reduced *H. khalidbinsultanii* to a synonym under *H. lodarensis*; here, the two taxa are differentiated according to the shape of the papillae and the corolla (see the key in the next section). In sect. Huernia, the shape of the papillae provides an important character when it is combined with other characters, such as inner corona and the shape of the corolla is the most important character indicative of the relationship between species (Leach 1983). Thus, this study suggests that *H. khalidbinsultanii* must be maintained.

*Huernia collenetteae* and *H. saudi-arabica* accessions overlapped in one cluster in both the UPGMA (Fig. 3) and PCoA (Fig. 3) analyses. Close examination of the relevant specimens shows that characters of *H. saudi-arabica* are accommodated within the variation range of *H. collenetteae*. The most noticeable character in Plowes (2012), which can be used to distinguish them, is patterns of streaking on the inside of the corolla (see figs 1–6 in Plowes 2012). Inside, the corolla tube in *H. saudi-arabica* (Jabal Sawdah population) is uniformly coloured with purple and with deeply coloured areas concentrated between the lobes; sometimes the inside of the corolla is entirely purple (Fig. 10 D, H, K). However, this pattern has also been seen in the population of *H. collenetteae* from Ash Shafa region (from 379.87 km north of Jabal Sawdah) (Fig. 10E, I; *S.A. Alharbi S4B*), but with concentric broken maroon lines and stripes in the corolla tube instead of a uniform maroon colour. Colours vary greatly in the flowers of widespread species of sect. Huernia, such as that recorded in *H. thuretii* and *H. hallii* from South Africa (Bruyns 2005). Thus, separating these two entities (*H. collenetteae* and *H. saudi-arabica*) into distinct species is inconsistent and this study will handle all data obtained from specimens of *H. collenetteae* as *H. saudi-arabica* in the subsequent discussion.

*Huernia saudi-arabica* (Fig. 10D), described from a single specimen *Collenette* 549, was related by its author to *H. lodarensis* and *H. boleana* (Field 1980). Distinctive features given are: 1) the corolla is slightly larger; 2) the corolla lobes have a more conspicuous papillose, frill-like margin; 3) the inner surface of the attenuate lobe-tip is covered with short, but even-sized, papillae; 4) the outer corona is distinctly 5-lobed, but, unlike *H. lodarensis*, each lobe is considerably wider than long and narrows towards the bifid tip rather than being somewhat parallel-sided; and 5) the inner corona-lobes are smooth and more acute towards the tips (fig. 1 in Field 1980). The floral measurements given by Field for *H. lodarensis* and *H. boleana* are easily accommodated within the known range of *H. saudi-arabica*. In the case of the third point, Field stated that “in *H. lodarensis*, the indumentum is a mixture of a few papillae and low tubercles” (fig. 1K in Field 1980). This feature can clearly be seen in a number of samples of *H. saudi-arabica*. In the case of the last two points, the corona lobes turned out to be far more variable than suspected and the range, that was observed, was found in specimens collected at a single locality. Here, it was found that the outer corona lobes range from rectangular to subquadrate or rarely fused entirely to form a disc (e.g. *Alharbi S5B*). Likewise, the tips of the inner coronal lobes vary from smooth to minutely scabrous. Consequently, our results support Bruyns’ (2005) opinion that *H. saudi-arabica* should treated as a synonym under *H. lodarensis* (Fig. 10A).

The numerical analysis carried out in this study did not resolve the relationship between *H. lodarensis*, *H. boleana*, *H. foetida* and *H. rubrosticta* and the other species. This is probably due to the low number of specimens included in the analysis and the incomplete nature of the data obtained from literature for *H. lodarensis* and *H. boleana*. However, a thorough examination of *H. foetida* (Fig. 12) and *H. rubrosticta* (Fig. 14B) type specimens reveal that they are very close to *H. lodarensis*. In view of the unique streaking patterns on the interior of the corolla that were not observed in any specimens of *H. lodarensis*, these taxa are described here as varieties under *H. lodarensis*. Nevertheless, many more samples and additional taxonomic work are considered necessary to either confirm or modify this treatment.

**Figure 4.**
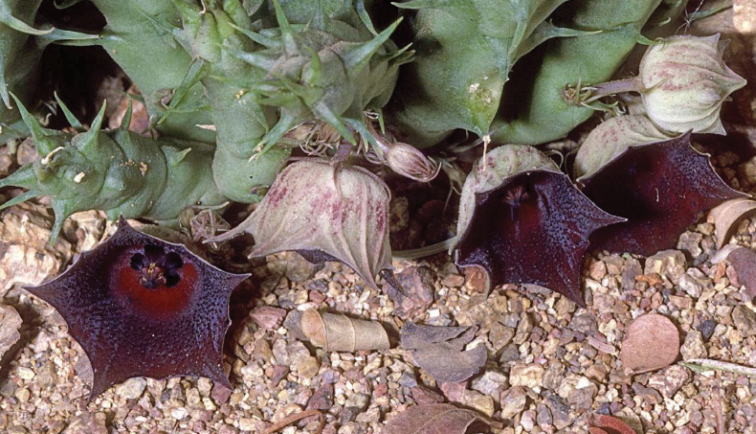
*Ceropegia macrocarpa*, Jabal Melhan, 20 km E Al Mighlaf, Yemen, ex JRI Wood 1202, sub DP7571. Reproduced from Plowes (2014).

The delimitation of *H. arabica* (Fig. 4) is a matter of long debate. While Plowes (2014) considered the taxon as an accepted species, Albers and Meve (2002) and Bruyns (2005) considered it to be synonymous under *H. macrocarpa* and *H. penzigii*, respectively. On the other hand, Berger (1910) and White and Sloane (1937) considered the taxon to be a variety under *H. penzigii* and *H. macrocarpa*, respectively. As not enough samples were available for this study to decide whether the species with uniformly purplish-maroon flowers (*H. arabica*, *H. macrocarpa* and *H. penzigii*) are synonymous or distinct species, Albers and Meve (2002) were followed. They treated *H. arabica* and *H. penzigii* as synonymous under *H. macrocarpa*.

In order to know the extent of variability within taxa, examining as many samples as possible is crucial. Our results show that the major weakness of previous taxonomic accounts of the Arabian members of sect. Huernia, particularly in Saudi Arabia, was caused by the fact that the taxa were described from single or only very small numbers of plants. This led to the recognition of many unnatural taxa, as seen in some of Plowes’ (2012, 2014) names. The ‘folk concept’ of species (Cronquist 1988), in which groups are formed intuitively by individuals ‘essentially similar’ and referred to as species, are found in the taxonomy of most succulent plants and largely held sway amongst the stapeliads (Bruyns 2005). This can clearly be seen in Leach’s (1988) taxonomic revision of *Huernia*. As he saw relatively few specimens, this led him to recognise too many taxa (64) and his classifications turned out to have little predictive value (Bruyns 2005). Thus, dense sampling of sect. Huernia or of stapeliads in general is essential, especially when describing new species or assessing the status of species. Members of sect. Huernia in Saudi Arabia still need attention and, with the aid of modern molecular methods, it seems that their complexity can be mastered. Our results suggest reducing the number of names reported in Saudi Arabia from 11 to four species: *H. khalidbinsultanii*, *H. laevis* and *H. lodarensis* (including three proposed varieties: var. lodarensis, var. foetida and var. rubrosticta) and *H. macrocarpa*.

Based on recent phylogenetic reconstructions in the Ceropegieae, the species of *Huernia* were transferred to *Ceropegia*, where they were placed under sect. Huernia and over 50 new combinations were made (Bruyns et al. 2017). However, Plowes’ names of Saudi Huernias are still not transferred yet. Therefore, we propose three new combinations in Ceropegia sect. Huernia. Those are: *C. khalidbinsultanii* comb. nov., C. lodarensis var. foetida comb. nov. and C. lodarensis var. rubrosticta comb. nov.

## Taxonomic treatment

### Ceropegia sect. Huernia

Taxon classificationPlantaeGentianalesApocynaceae

(R.Br.) Bruyns, S. African J. Bot. 112: 423 (2017).

80CAB4B7-8B34-57ED-9C37-2DC9BE734681

≡ Huernia R.Br., Mem. Wern. Nat. Hist. Soc.: 22 (1810). Lectotype
Huernia campanulata (Masson) Haw. (designated by White and Sloane (1937))
= Ceropegia clavigera (Jacq.) Bruyns.

#### Diagnostic features.

Perennial leafless dwarf succulent herb, mat-forming rarely rhizomatous, sometimes prostrate or pendulous succulent. ***Branches*** glabrous, smooth, 4- to 16-angled. ***Leaves*** reduced mainly to soft point without stipular structures. The leaf-rudiments are borne on a raised tubercle which is a much swollen leaf-base. These ***tubercles*** are arranged in rows along the branch and joined towards their bases into angles along the branch with a groove between vertical rows of tubercles. ***Inflorescence*** glabrous, usually only one per branch, arising mainly in lower half of branch between tubercles, 1–10 flowered. ***Corolla*** urceolate to campanulate to subrotate, shallowly lobed. ***Staminal corona*** in two well-separated series, inner pressed to backs of anthers mostly exceeding them and meeting in centre, often with prominent transversely-rounded dorsal projections. Outer spreading along base of tube, discrete to fused into spreading disc with fleshy tubercle beneath guide-rail obscuring entrance to small nectarial cavity. ***Anthers*** horizontal on top of style-head, margins shrinking back to expose pollinia, rectangular. ***Pollinium*** ellipsoidal, longer than broad, insertion-crest exactly along outer edge, caudicle attached with broad cupular pad to base. ***Follicles*** erect, terete-fusiform, obclavate, slender, consisting of two horns diverging at 30–60°, longitudinally mottled with narrow broken purple stripes, glabrous, smooth (Bruyns 2005, 2014; Bruyns et al. 2017).

### Key for Ceropegia sect. Huernia in Saudi Arabia

**Table:** 

1	Corolla inside glabrous or covered with papillae ≤ 1 mm long; inner corona lobes not or shortly exceeding anthers, not tapering to a fine point	**2**
–	Corolla inside densely covered with papillae > 1 mm long; inner corona lobes much exceeding anthers, tapering to a fine point	**3**
2	Corolla bowl-shaped, papillate, uniformly purplish-maroon without annulus around mouth of tube	***C. macrocarpa***
–	Corolla funnel-shaped, glabrous, shiny with irregular broad red streaks on a yellowish background with an annulus-like structure around mouth of tube	***C. laevis***
3	Tubercles on branches up to 16 mm long; corolla covered with slender (hair-like) papillae, tubular-campanulate, lobes ascending	***C. khalidbinsultanii***
–	Tubercles on branches up to 12 mm long; corolla covered with conical compressed papillae, campanulate, lobes spreading or reflexed	***C. lodarensis***

### 1. Ceropegia macrocarpa

Taxon classificationPlantaeGentianalesApocynaceae

(Sprenger) Bruyns, S. African J. Bot. 112: 424 (2017)

D346054A-D9DE-5F18-8F4A-81FCF7199439

urn:lsid:ipni.org:names:77215098-1

Figs 4 , 5 ; Map 1

≡ Huernia macrocarpa Sprenger, Cat. Dammann & Co. 59: 4 (1892) Type: Eritrea • Penzig s.n. (K epitype).
= Huernia macrocarpa var. arabica (N. E. Brown) A. C. White & B. Sloane (1937)
= Huernia macrocarpa var. penzigii (N. E. Brown) A. C. White & B. Sloane (1937)
= Huernia macrocarpa var. schweinfurthii (A. Berger) A. C. White & B. Sloane (1937)
= Huernia penzigii N. E. Brown (1892)
= Huernia penzigii var. arabica (N. E. Brown) A. Berger (1910)
= Huernia penzigii var. schimperi A. Berger (1910)
= Huernia penzigii var. schweinfurthii A. Berger (1910)

#### Description.

Dwarf succulent forming dense clump. ***Branches*** 60 mm long, non-rhizomatous, erect, decumbent, grey-green mottled with purple-red; tubercles up to 10 mm long (including leaf-rudiment), conical, spreading, laterally flattened and joined into 5 angles along branch, each tipped with a soft slender acuminate caducous leaf-rudiment. ***Inflorescence*** usually only 1 per branch, arising in lower half of branch, each bearing 2–3 flowers developing mainly successively, flowers with no unpleasant smell; ***pedicel*** spreading and holding flower facing horizontally. ***Corolla*** 15 mm diam., shallow bowl shape; outside smooth, cream-speckled with maroon, with 1 heavy (+ 2 lighter) raised longitudinal veins running from lobes to base of tube; inside uniformly coloured with purplish-red, covered except in lower third of tube with very small wart-like papillae; ***tube*** cupular; ***lobes*** ascending, deltoid, acuminate. ***Corona*** without basal stipe; ***outer lobes*** spreading on base of tube and fused partially to it, discrete to 5-lobed with each lobe subquadrate emarginate or slightly crenate, blackish-maroon; ***inner lobes*** maroon, adpressed to backs of anthers exceeding them and meeting in centre, dorsiventrally flattened around laterally broadened base becoming terete above and tapering gradually to obtuse bristly apex, a transversely conspicuously gibbous, broadened at the base with an acute end.

**Map 1.**
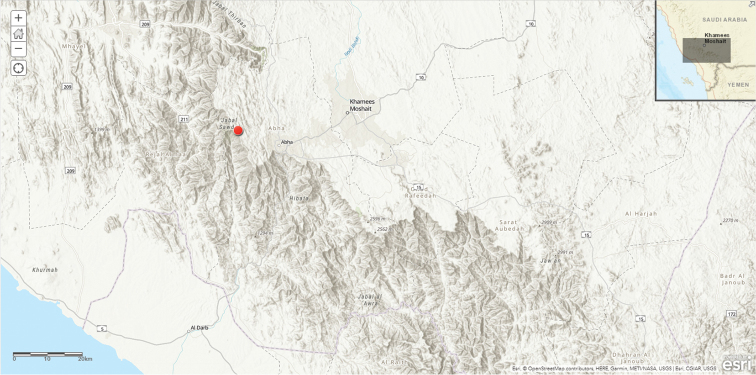
Distribution of *Ceropegia macrocarpa* in Saudi Arabia.

#### Distribution in Saudi Arabia.

Rare, known only from Asir between Abha and Jabal Sawdah, SW Saudi Arabia (Chaudhary 2001).

#### General distribution.

Somaliland, Sudan, Eritrea, Ethiopia, South west Arabian Peninsula (Saudi Arabia, Yemen) (Albers and Meve 2002).

#### Habitat and ecology.

Growing amongst granitic rocks and scattered shrubs on a steep hillside at 2700 m alt. (Collenette 1999).

#### Diagnosis.

This species can be easily distinguished from other members of sect. Huernia in Saudi Arabia by the small maroon bowl-shaped flowers.

#### Etymology.

Macrocarpus (Greek) 'makros', large; and 'karpos', fruit (Eggli and Newton 2004).

#### Preliminary conservation status.

The species is known only from one location near Sawda Mountain; the estimated EOO and AOO of 8 km^2^ would place the species in the Critically Endangered (CR) status. However, little is known about the size of the population and possible threats. Therefore, Data Deficient (DD) is assigned to this species.

**Figure 5.**
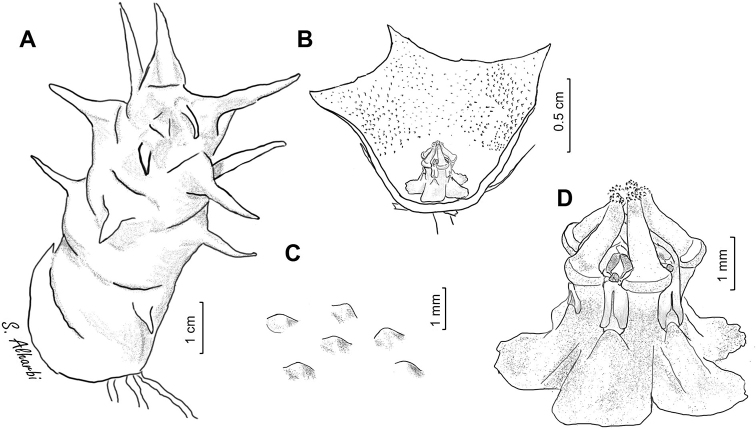
*Ceropegia macrocarpa***A** branch **B** side view of dissected flower **C** papillae inside corolla in mouth of tube **D** side view of gynostegium. Drawn from photo of ex JRI Wood 1202, sub DP7571, Jabal Melhan, 20 km E Al Mighlaf, Yemen.

#### Additional specimens examined.

Ethiopia, *Gilbert 2945* (E [fl in spirit])

### 2. Ceropegia khalidbinsultanii

Taxon classificationPlantaeGentianalesApocynaceae

(Plowes & McCoy) Alharbi & Al-Qthanin comb. nov.

A151677B-F9C2-5E28-A3C1-4C0D8A388272

urn:lsid:ipni.org:names:77215099-1

Figs 6 , 7 ; Map 2

≡ Huernia khalidbinsultanii Plowes & McCoy, Cact. Succ. J. (Los Angeles) 75(1): 19 (2003). Type: Saudi Arabia – Asir • *T.A. McCoy 2446* (holotype: MO; isotypes P & SRGH); 25 km SW of Khamis Mushayt; 18°4.0906'N, 42°43.8908'E; alt. 2100 m; 15 Jan 1999.
= Huernia asirensis Plowes, Asklepios 114: 7 (2012), syn. nov. Type: Saudi Arabia – Tanumah • *I.S. Collenette 2655* (Holotype: K!, [fl in spirit: 44279.000]); 12 km S. of An Numas on Taif to Abha Road;18°56.1481'N, 42°11.2139'E; alt.1800 m; 10 May 1981.
= Huernia radhwana Plowes, Asklepios 114: 10 (2012), syn. nov. Type: Saudi Arabia – Jabal Radhwa • *I.S. Collenette 5944* (Holotype: K!, [fl in spirit: 51187.000]); 75 km NE Yanbu; 24°32.3717'N, 38°20.4741'E; alt. 1750 m; 01 Feb 1987.
= Huernia anagaynensis Plowes, Asklepios 114: 7 (2012), syn. nov. Type: Saudi Arabia – Jabal Anagayn • *I.S. Collenette 5970* (Holotype: K!, [fl in spirit: 50937.000]); 95 km south of Madinah; 23°21.5747'N, 39°34.9766'E; alt. 1371 m; 06 Dec1986.
Huernia haddaica Al-Hemaid (*nom. inval*. Art 37.2), Saudi J. Biol. Sci. 8: 168 (2001).

#### Description.

Dwarf succulent forming dense clump. ***Branches*** 50–100 mm long, non-rhizomatous, decumbent, grey-green mottled with purple-red; tubercles 7–16 mm long (including leaf-rudiment), 1.5–5 mm broad at base, conical, spreading, laterally flattened and joined into 5 angles along stem, each tipped with a soft slender acuminate caducous leaf- rudiment. ***Inflorescence*** usually only 1 per branch, arising in lower half of branch, each bearing 1–7 flowers developing mainly simultaneously or in gradual succession from short peduncle, with several filiform bracts without lateral teeth, flowers with mainly very foetid odour, rarely faint or no unpleasant smell; ***pedicel*** 5–21.5 mm long, 1–2 mm thick, spreading and holding flower facing horizontally, tapering sometimes toward the point of flower attachment; ***sepals*** 10–18 mm long, 1–2 mm broad at base, attenuate. ***Corolla*** 27–47 mm diam., tubular-campanulate to campanulate; outside smooth, white to creamy-white or cream speckled with pale maroon spots uniformly scattered, sometimes spots become darker especially on the lower or upper half of corolla tube, with 1 heavy (+ 2 lighter) raised longitudinal veins running from lobes to base of tube; inside creamy-white to cream with irregular-shaped narrow short maroon lines and dashes changing to narrow concentric broken lines in lower half of tube, covered except in lower third of tube with slender (hair-like) papillae densely crowded around mouth of tube (up to 3 mm long and 0.75 mm an base in tube mouth), each tipped by minute apical acuminata bristle; ***tube*** 7–13 mm long, 9–14 mm broad at mouth, pentagonal; ***lobes*** 13–18 mm long, 8–12 mm broad at base, ascending to slightly spreading, narrowly deltoid and usually longer than wide, attenuate usually concave or form channel above, ***intermediate lobes*** 1–2.5 mm long. ***Corona*** without basal stipe; ***outer lobes*** (4.5–8 mm diam.) spreading on base of tube and fused partially to it; fused together into disc with crenate margin to a slightly disc-like with rounded to subquadrate short and broad lobes, rarely discrete to 5-lobed with each lobe subquadrate mucronate, blackish maroon; ***inner lobes*** 3–5.5 mm long, 0.5–1.5 mm at base, ivory white sometimes mottled with a few maroon spots at the tips, adpressed to anthers in their lower half then rising up connivent and then diverging towards apices, dorsiventrally flattened around laterally-broadened base becoming terete above and tapering gradually to a slender fine minutely-scabrous apex, at base with inflated transversal dorsal gibbosity with rounded to acute end. ***Pollinia*** 0.1–0.7 mm long.

**Map 2.**
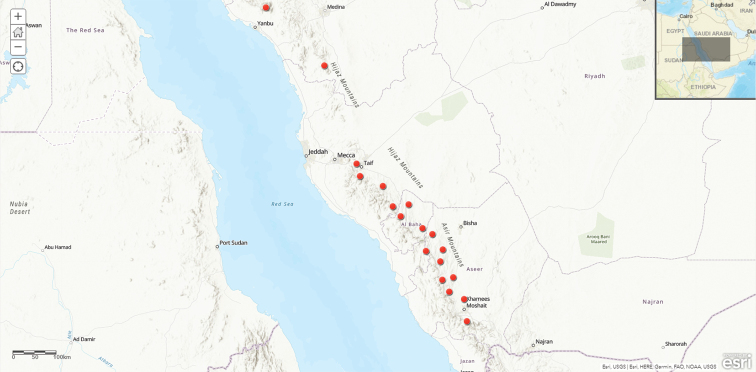
Distribution of *Ceropegia khalidbinsultanii*.

#### Distribution in Saudi Arabia.

Scattered over a wide area, extending from Khamis Mushait in SW of the country to Jabal Radhwa, 75 km north of Yanbu in the Western Region.

#### General distribution.

Probably endemic to SW Arabian Peninsula, known so far from Saudi Arabia only.

#### Habitat and ecology.

Growing on granitic outcrops often under shrubs, from 1800–2100 m alt. Flowering Dec.-May

#### Diagnosis.

*Ceropegia khalidbinsultanii* is best distinguished from the closely-related *C. lodarensis* by longer tubercles (up to 16 mm) on the branches and the smaller, white to creamy-white tubular-campanulate corolla streaked with narrow maroon lines, slender (hairy) papillae and a very foetid odour.

#### Notes.

The foetid odour of the flower becomes weak or completely vanishes after all pollinia have been removed from the flower.

#### Etymology.

Khalidbinsultanii for Prince Khalid bin Sultan bin Abdulaziz M., a former Saudi Deputy Minister of Defence (Plowes and McCoy 2003).

#### Preliminary conservation status.

Near Threatened (NT) has been assigned to *Ceropegia khalidbinsultanii*, based on the species’ EOO of 41,490 km^2^ and AOO of 2,012 km^2^ and the current threats of habitat transformation (roads and housing construction), population fragmentation and tourism.

**Figure 6.**
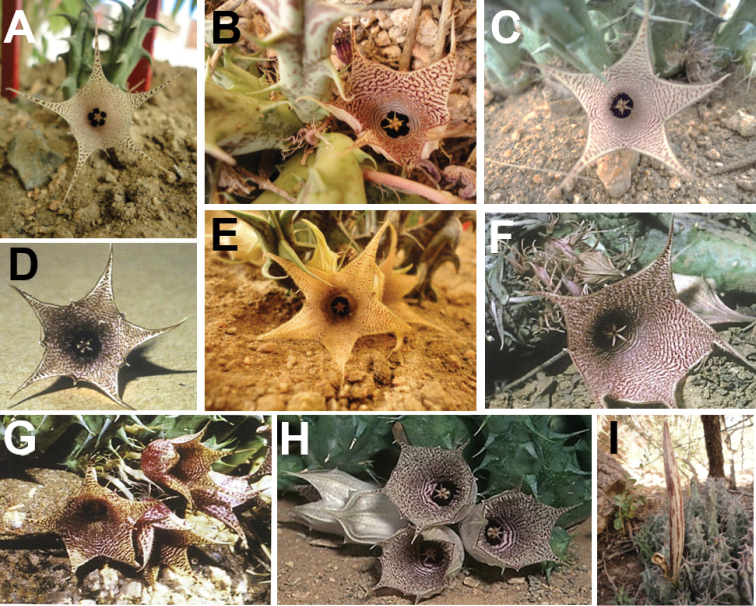
*Ceropegia khalidbinsultanii***A***Alharbi S00* (*H. asirensis*) **B***Alharbi S10b* (*H. asirensis*) **C***Alharbi S4a* (*H. asirensis*) **D***Collenette 5944*, Jabal Radhwa, (*H. radhwana*, Type) **E***Alharbi S16a* (*H. asirensis*) **F***Collenette 1309*, Al-Baha, (*H. asirensis*, Type) **G***Collenette 5970*, Jabal Anagyan (*H. anagaynensis*, Type) **H** ex *Tom McCoy KSA129 sub DP8384*, 25 km SW of Khamis Mushayt, (*H. khalidbinsultanii*, Type) **I** follicles, *Alharbi S14a*. (**A–C, E, I**) photo by the first author from Wadi Thee Gazal, Ash Shafa; (**D, F, G**) reproduced from Plowes (2012)); (**H**) received from D. Plowes in 2011.

#### Additional specimens examined.

Saudi Arabia – Al-Taif • *S.A. Alharbi S3a* (UQU); Wadi Thee Gazal, Ash Shafa; 21°5.5702'N, 40°21.785'E; alt. 2057 m; 23 Jan 2011; *S.A. Alharbi S4a* (UQU); same data as for preceding; 1 Jan 2011; *S.A. Alharbi S6a* (UQU); same data as for preceding;10 Jan 2011; *S.A. Alharbi S7a* (UQU); same data as for preceding; 9 Dec 2010; *S.A. Alharbi S8a* (UQU); same data as for preceding; 19 Jan 2011; *S.A. Alharbi S16a* (UQU); same data as for preceding; 8 Jan 2011; *S.A. Alharbi S16A* (UQU); same data as for preceding; 30 Dec 2010; *S.A. Alharbi S13a* (UQU); same data as for preceding; 9 Dec 2010; *S.A. Alharbi S14a* (UQU); same data as for preceding; 17 Dec 2010; *S.A. Alharbi S00* (UQU); 9 Dec 2010; *S.A. Alharbi S10b* (UQU); same data as for preceding; 21°5.4656'N, 40°21.7937'E; 9 Dec 2010.

**Figure 7.**
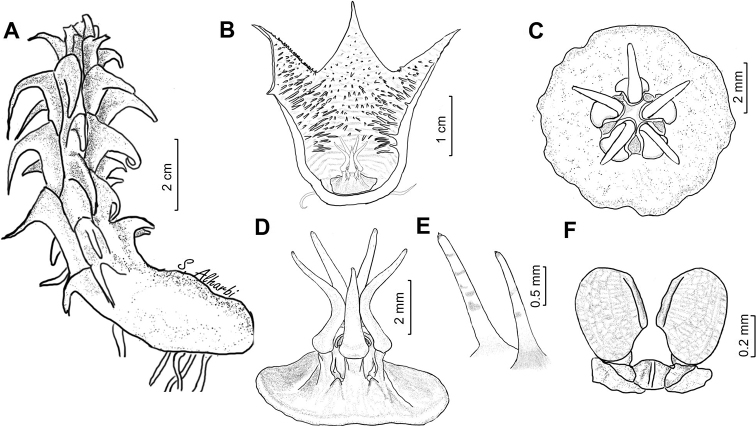
*Ceropegia khalidbinsultanii***A** branch **B** side view of dissected flower **C** face view of gynostegium **D** side view of gynostegium **E** papillae inside corolla in mouth of tube **F** pollinarium. Drawn from *S.A. Alharbi S4a*, Wadi Thee Gazal, Ash Shafa.

### 3. Ceropegia laevis

Taxon classificationPlantaeGentianalesApocynaceae

(J.R.I. Wood) Bruyns, S. African J. Bot. 112: 424 (2017)

174010D0-03D5-5A68-8B22-5F861CBA273B

Figs 8 , 9 ; Map 3

≡ Huernia laevis J.R.I. Wood, Kew Bull. 39:128 (1984).

#### Type.

Yemen • *J.R.I. Wood 3037* (***holotype***: K [46740.000]); Jebel Marran, Khawlan As Sham; 16°49.2672'N, 43°24.7619'E; alt. 1400 m; 31 Oct 1979.

#### Description.

Dwarf succulent forming dense clump. ***Branches*** non-rhizomatous, up to 80 mm long, erect, decumbent, grey-green mottled with purple or red; tubercles 3–5 mm long, 1 mm broad at base, conical, spreading, laterally flattened and joined towards base into 5 angles along branch, abruptly narrowing into fine spreading slender acuminate tooth. ***Inflorescences*** 1–2 per branch, each of 2–5 flowers developing in gradual succession on short peduncle with few narrow filiform bracts; ***pedicel*** 15 mm long, spreading and holding flower facing horizontally; flowers with no scent; *sepals* 15 mm long, 3 mm broad at base, narrowly ovate attenuate. ***Corolla*** 32 mm diam., broadly funnel-shaped, margin weakly bulging like an annulus; outside smooth, pale cream with 1 heavy (+ 2–4 lighter) raised longitudinal veins running down each lobe; inside shiny creamy-yellow, marked with shiny irregular broad maroon streaks and scrolls, smooth with few low conical papillae (wart-like) at corolla lobes apices each with minute apical bristle; *tube* 6 mm long, 10 mm broad at mouth, cupular; ***lobes*** 10 mm long, 14 mm broad at base, reflexed, deltoid-acuminate, ***intermediate lobes*** 1 mm long. ***Corona*** without basal stipe; ***outer lobes*** (discrete 5 lobes), 4 mm diam., subquadrate, emarginate to shallowly bifid, spreading on base of tube and fused to it towards base, blackish-maroon; *inner lobes* 1 mm long, purple with cream at base, adpressed to backs of anthers and shorter than them, dorsiventrally flattened with ascending obtuse conspicuous gibbous at base, tapering to small smooth acute apex.

**Map 3.**
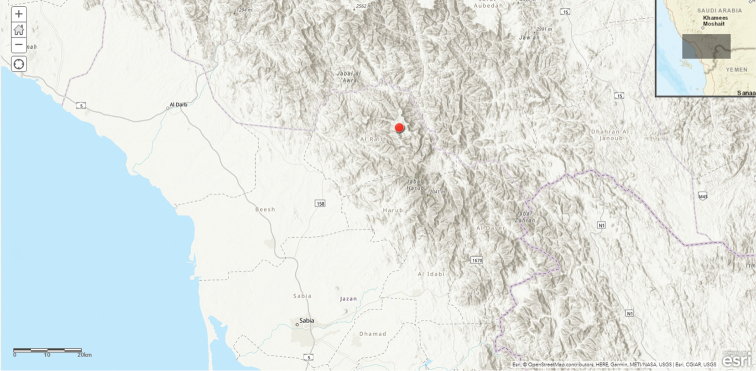
Distribution of *Ceropegia laevis* in Saudi Arabia.

#### Distribution in Saudi Arabia.

Jabal Al Qahar, 90 km NE of Baysh, Jazan, SW Saudi Arabia (Chaudhary 2001).

#### General distribution.

Probably endemic to SW Arabian Peninsula, known so far from Saudi Arabia and Yemen (Chaudhary 2001).

#### Habitat and ecology.

Growing amongst limestones amongst *Juniperus* at 1828–2000 m alt. (Collenette 1999). Flowering: mainly September–May

#### Preliminary conservation status.

*Ceropegia laevis* should be considered as Nationally Endangered (EN), according to the IUCN Red List criteria. The species is known from only one location, its EOO and AOO (104.00 km^2^) would both qualify as Endangered. Its habitat is not part of any protected area and its continuing decline is projected because of anthropogenic activities in the area.

**Figure 8.**
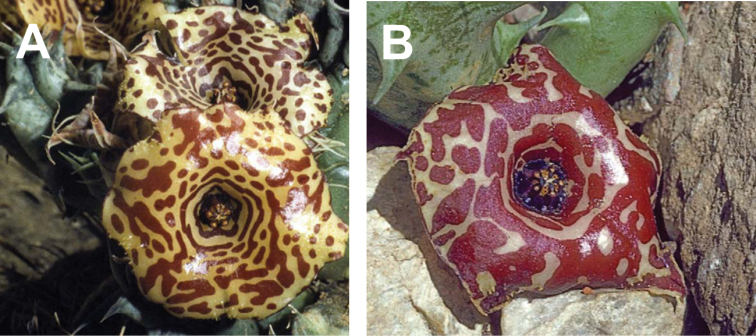
*Ceropegia laevis*, Jabal Qahar, 90 km NE of Baysh, Jazan, Saudi Arabia **A***Collenette 8177***B** ex *S Collenette 8180* sub *DP8296*. Reproduced from Plowes (2014).

#### Diagnosis.

*Ceropegia laevis* can easily be distinguished from most other species of sect. Huernia in Saudi Arabia by the glabrous shiny yellow background colour of the inside of the corolla, which has an annulus-like area around the mouth of the tube.

#### Etymology.

Laevis (Latin) smooth, flat; for the glabrous corolla (Eggli and Newton 2004).

#### Specimens examined.

Saudi Arabia – Jazan • *I.S. Collenette 8180* (K [fl in spirit: 57656.000]); Jabal Qahar; 17°42.0367'N, 42°51.1983'E; alt. 2000 m; 20 Apr 1992.

**Figure 9.**
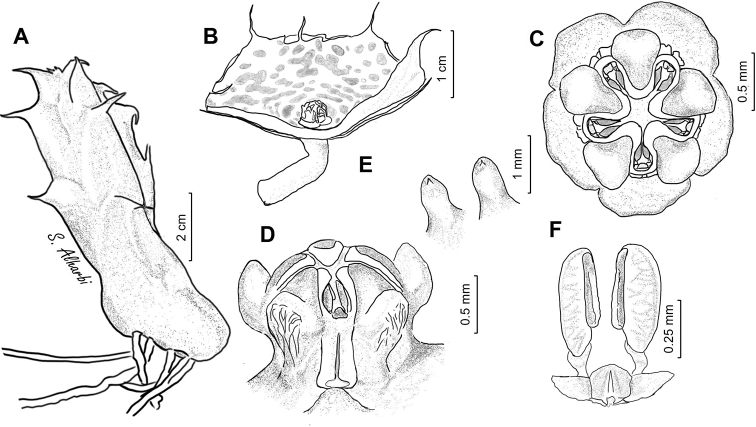
*Ceropegia laevis***A** branch **B** side view of dissected flower **C** face view of gynostegium **D** side view of gynostegium **E** papillae inside corolla in lobe tip **F** pollinarium. Drawn from *Collenette 8180*, Jabal Qahar.

### 4. Ceropegia lodarensis

Taxon classificationPlantaeGentianalesApocynaceae

(Lavranos) Bruyns, S. African J. Bot. 112: 424 (2017).

946BBC3D-1EF5-5458-9529-D65BA37E783F

#### Description.

Dwarf succulent forming dense clump. ***Branches*** 30–100 mm long, non-rhizomatous, erect to decumbent, grey-green mottled with purple-red; tubercles 4–10 mm long (including leaf-rudiment), 1.5–5 mm broad at base, conical, spreading, laterally flattened and joined into 5 angles along branch, each tipped with a soft slender acuminate caducous leaf-rudiment. ***Inflorescence*** arising in lower half of branch, usually 1 per branch, each bearing 2–10 flowers developing in gradual succession from short peduncle sometimes 3 flowers developing simultaneously, bracts filiform without lateral teeth, flowers with no foetid odour, rarely with faint unpleasant smell; ***pedicel*** 8–20 mm long, 1–2.5 mm thick, ascending holding flower facing upwards, tapering sometimes toward the point of flower attachment; ***sepals*** 8–18 mm long, 1–2.5 mm broad at base, attenuate. ***Corolla*** 30–50 mm diam., campanulate; outside smooth, cream-speckled with pale maroon spots uniformly scattered or concentrated on the upper half of corolla tube sometimes spots become darker especially on the upper half, with 1 heavy (+ 2–4 lighter) raised longitudinal veins running from lobes to base of tube; inside cream with irregular-shaped short maroon lines and dashes changing to concentric broken lines in lower half of tube or uniformly coloured with purplish-red, sometimes deep coloured areas concentrated between the lobes or corolla entirely uniformly coloured with purplish-red; corolla covered, except in lower third of tube with compressed conical papillae densely crowded and reaching maximum size around mouth of tube (up to 3 mm long and 1.2 mm base width), each tipped by minute apical acuminata bristle; ***tube*** 7.5–15.5 mm long, 11–22 mm broad at mouth, pentagonal; ***lobes*** 9–22.8 mm long, 9–14.25 mm broad at base, spreading with recurved apex or sometimes reflexed, deltoid, caudate to acute or acuminate rarely attenuate usually concave or form channel at tip, ***intermediate lobes*** 1.5–4 mm long. ***Corona*** without basal stipe; ***outer lobes*** (5–10 mm diam.) spreading on base of tube and fused partially to it, discrete to 5 lobes with each lobe rectangular rarely subquadrate crenate, dentate, mucronate, emerginate or bifid blackish-maroon; ***inner lobes*** 3–6 mm long, 1–1.5 mm at base, ivory white sometimes mottled with a few maroon spots at the tips or marked entirely with small purple spots adpressed to anthers in their lower half, then rising up connivent and then diverging towards apices, dorsiventrally flattened around laterally-broadened base becoming terete above and tapering gradually to a slender fine minutely-scabrous or smooth apex, at base with slightly inflated transversal dorsal gibbosity with rounded to truncate end, sometimes a conspicuous acute humb appearing in the staminal tube under corona base, rarely hook-like appendages grow from both sides of the base meeting above the guardrails. ***Pollinia*** 0.7–0.8 mm long.

### Key to three varieties of *Ceropegia lodarensis*

**Table:** 

1	Branches not stout, up to 100 mm long; corolla up to 50 mm diam., marked inside with irregular short maroon lines and dashes or rarely dotted; outer corona mostly consisting of five distinct rectangular lobes	**2**
–	Branches stout, up to 60 mm long; corolla up to 30 mm diam., marked inside with maroon rounded spots or dashes; outer corona disc or disc-like	**C. lodarensis var. rubrosticta**
2	Corolla tube inside with concentric broken lines or uniformly coloured with purplish-red, lobes marked with irregular-shaped short maroon lines and dashes sometimes deep coloured areas concentrated between the lobes or corolla entirely uniformly coloured with purplish-red	**C. lodarensis var. lodarensis**
–	Corolla tube inside with concentric short dashes, corolla lobes marked with small maroon dots	**C. lodarensis var. foetida**

### 4.1 Ceropegia lodarensis (Lavranos) Bruyns var. lodarensis

Taxon classificationPlantaeGentianalesApocynaceae

AE992C49-362B-50AF-9065-39895C663D6F

**Map 4.**
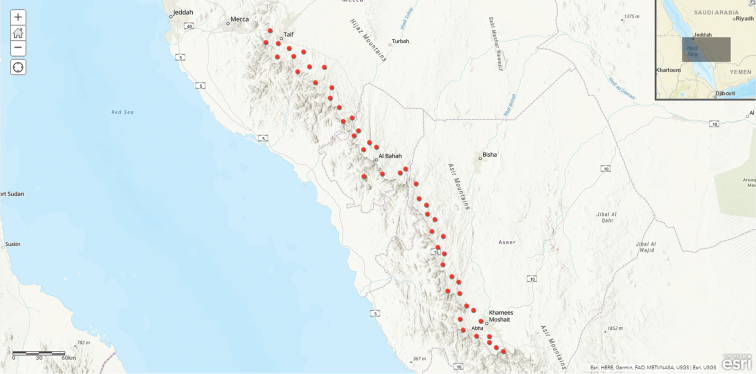
Distribution of Ceropegia lodarensis var. lodarensis in Saudi Arabia.

**Figure 10.**
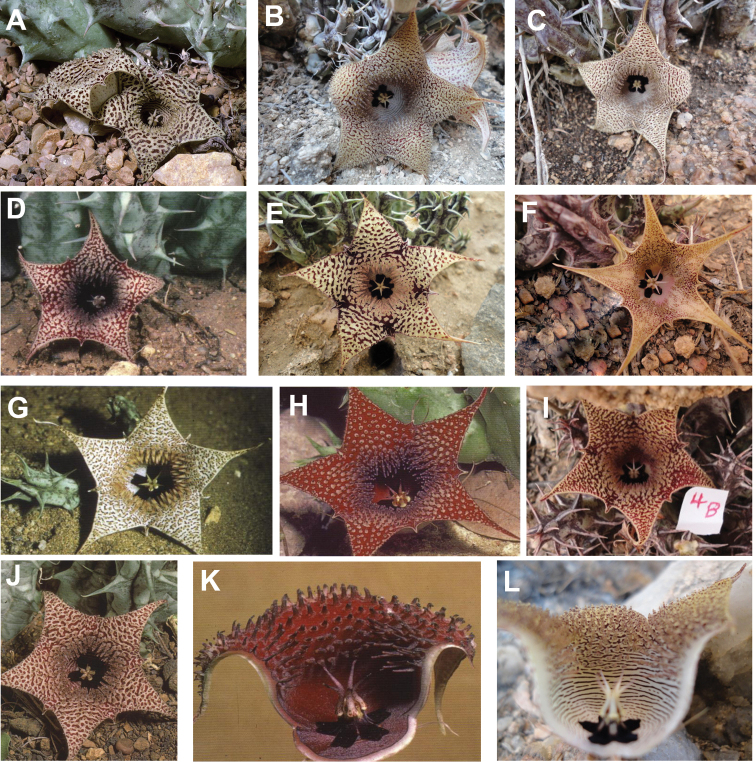
Ceropegia lodarensis var. lodarensis**A** ex *J Lavranos 1789*, sub *DP3604*, Yemen, (*H. lodarensis*, Type) **B***Alharbi S6B* (*H. collenetteae*) **C***Alharbi S9B* (*H. collenetteae*) **D** ex *Collenette 549* sub *DP6865*, Jabal Al Sawdah, (*H. saudi-arabica*, Type) **E***Alharbi S2B* (*H. collenetteae*) **F***Alharbi S18a* (*H. collenetteae*) **G***Collenette 2227*, Al-Hadda, (*H. collenetteae*) **H** ex *Collenette 8232* sub *DP8126*, (*H. saudi-arabica*) **I***Alharbi S4B* (*H. collenetteae*) **J** ex *Collenette 1176* sub *DP6868*, Jabal Al Sawdah, (*H. collenetteae*, Type) **K** maroon uniform colour of corolla tube in ex *Collenette* sub *DP6594*, Abha, (*H. saudi-arabica*) **L** concentric broken maroon lines of corolla tube in *Alharbi S6B* (*H. collenetteae*). (**A**) reproduced from Plowes (2014); (**D, G, H, J, K**) reproduced from Plowes (2012); (**B, C, E, F, I, L**) photo by the first author from Wadi Thee Gazal, Ash Shafa.

**Figure 11.**
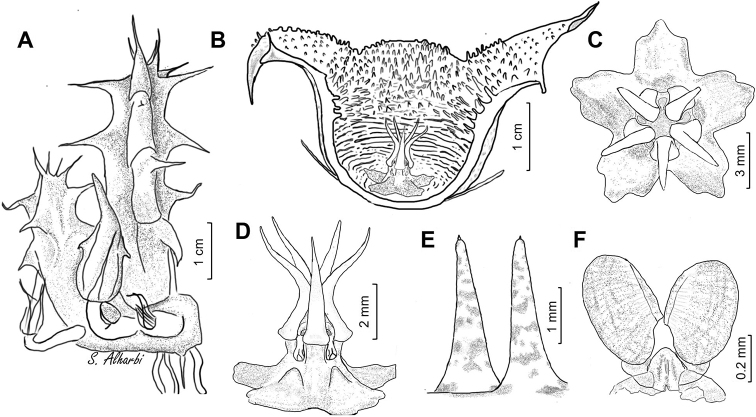
Ceropegia lodarensis var. lodarensis**A** branch **B** side view of dissected flower **C** face view of gynostegium **D** side view of gynostegium **E** papillae inside corolla in mouth of tube **F** pollinarium. Drawn from (**A**) *S.A. Alharbi S2B*; (**B–F**) *S.A. Alharbi S6B*, Wadi Thee Gazal, Ash Shafa.

Figs 10 , 11 ; Map 4

≡ Huernia lodarensis Lavranos, J. S. African Bot. 30: 87 (1964). Type: Yemen – Lodar (Lawdar) • *J.J.Lavranos 1900* (holotype: K [fl in spirit: 24982.000]); 13°52.6751'N, 45°51.7598'E; alt. 900 m; 19 Aug 1962.
= Huernia collenetteae Plowes, Asklepios 114: 8 (2012). syn. nov. Type: Saudi Arabia – Asir • *I.S. Collenette 1176* (clonotype: SRGH [DP6868]); between Abha and Jabal Sawdah; 18°14.425'N, 42°25.2244'E; alt. 2650 m.
= Huernia saudi-arabica D.V.Field, Kew Bull. 35(4): 754 (1981). Type: Saudi Arabia – Asir • *I.S. Collenette 549* (holotype: k! [K000911103]); between Abha and Jabal Sawdah, 12 km NW Abha; 18°15.7389'N, 42°23.1535'E; alt. 2650 m.; 31 Mar 1978.

#### Description.

***Branches*** 30–90 mm long; tubercles 4–10 mm long (including leaf-rudiment), 1.5–5 mm broad at base. ***Inflorescence*** bearing 2–10 flowers developing in gradual succession from short peduncle, sometimes 3 flowers developing simultaneously, flowers with no foetid odour, rarely with faint unpleasant smell; ***pedicel*** 8–20 mm long, 1–2.5 mm thick, ascending holding flower facing upwards, tapering sometimes towards the point of flower attachment; *sepals* 8–18 mm long, 1–2.5 mm broad at base, attenuate. ***Corolla*** 30–50 mm diam., campanulate; outside smooth, cream-speckled with pale maroon spots uniformly scattered or concentrated on the upper half of corolla tube, sometimes spots become darker especially on the upper half; inside cream with irregular-shaped short maroon lines and dashes changing to concentric broken lines in lower half of tube or uniformly coloured with purplish-red, sometimes deep coloured areas concentrated between the lobes or corolla entirely uniformly coloured with purplish-red; ***papillae*** up to 3 mm long and 1.2 mm base width; ***tube*** 7.5–15.5 mm long, 11–22 mm broad at mouth, pentagonal; ***lobes*** 9–22.8 mm long, 9–14.25 mm broad at base, spreading to spreading with recurved apex or sometimes reflexed, deltoid, caudate to acute or acuminate, concave at tip, ***intermediate lobes*** 1.5–4 mm long. ***Outer corona lobes*** (5–10 mm diam.) discrete to 5 rectangular lobes; ***inner lobes*** 3–6 mm long, 1–1.5 mm at base.

#### Distribution in Saudi Arabia.

Scattered over a wide area, extending from Al Habala in SW of the country to Al-Hadda in Al-Taif in the Western Region.

#### General distribution.

Arabian Peninsula (Saudi Arabia and Yemen) and Africa (Ethiopia; *Bruyns*, *P.V. 8432*, E; http://data.rbge.org.uk/herb/E00995868)

#### Habitat and ecology.

It occurs at 900–2650 m alt. in granitic outcrops mainly under shrubs. Flowering: mostly Aug-May

#### Diagnosis.

Ceropegia lodarensis var. lodarensis is most similar to *C. khalidbinsultanii*, but differs in having a larger campanulate corolla with compressed conical papillae (up to 1.2 mm broad at base), sometimes uniformly coloured with purplish-red, flower with no or only faint bad smell and has shorter tubercles on the branches.

#### Etymology.

Lodarensis for the occurrence at Lodar (Lawdar) in Yemen (Eggli and Newton 2004).

#### Preliminary conservation status.

Ceropegia lodarensis var. lodarensis should be assessed as Near Threatened (NT) in Saudi Arabia due to species’ AOO of 3,900 km^2^ and EOO of 12,509.959 km^2^ and the current threats of tourism, overgrazing, infrastructure and housing development.

#### Additional specimens examined.

Saudi Arabia – Asir • *I.S. Collenette 1280* (k [fl in spirit: 44272.000] & E); Al Habala, 50 km SE of Abha; 18°1.6787'N, 42°51.3655'E; alt. 2384 m; 06 Apr1979.

Saudi Arabia – Al-Baha • *I.S. Collenette 7785* (k [fl in spirit: 57339.000]); Jabal Shada, SW of Al Baha; 19°50.9947'N, 41°19.0693'E; alt.1933 m; 07 Apr1991; *I.S. Collenette 8267* (K [fl in spirit: 59350.000]); same data as for preceding; 15 Sep1992.

Saudi Arabia – Al-Taif • *I.S. Collenette 815* (K!, herbarium specimen); Wadi Ammak near Al Hadda; 21°20.9808'N, 40°17.7485'E; alt. 2100 m; *I.S. Collenette 2227* (K [fl in spirit: 44371.000, 53692.000]); Al-Hada; 21°20.8387'N, 40°17.152'E; alt. 2000 m; 1981; *I.S. Collenette 2633* (K [fl in spirit:45473.000, 45894.000, herb. material sub DP6599 & sub Leach 17652]); SW of Al Hadda, off Taif to Abha Road; 21°18.4696'N, 40°22.1371'E; alt. 2100 m; 07 May 1981; *I.S. Collenette 5780* (K [fl in spirit: 35856.000]); Between Al Hadda and Harithi; 21°5.7571'N, 40°55.0155'E; alt. 1620 m; 23 Mar1986; *S.A. Alharbi S1b* (UQU); Wadi Thee Gazal, Ash Shafa; 21°5.4656'N, 40°21.7937'E; alt. 2057 m; 29 Dec 2010; *S.A. Alharbi S2b* (UQU); same data as for preceding; 09 Dec 2010; *S.A. Alharbi S3b* (UQU); same data as for preceding; 23 Jan 2011; *S.A. Alharbi S4b* (UQU); same data as for preceding; 29 Dec 2010; *S.A. Alharbi S5b* (UQU); same data as for preceding; 23 Nov 2010; *S.A. Alharbi S6b* (UQU); same data as for preceding; 02 Oct 2011; *S.A. Alharbi S9b* (UQU); same data as for preceding; 23 Nov 2010; *S.A. Alharbi S9a* (UQU); same data as for preceding; 05 Jan 2011; *S.A. Alharbi S18a* (UQU); same data as for preceding; 21°5.5702'N, 40°21.785'E; 17 Dec 2010.

*I.S. Collenette 1523* (E [fl in spirit]); *A.J. Bntler AJB 13* (E [fl in spirit]).

### 4.2 Ceropegia lodarensis (Lavranos) Bruyns var. foetida

Taxon classificationPlantaeGentianalesApocynaceae

(Plowes) Alharbi & Al-Qthanin comb. nov.

FA908D00-4A3C-5673-A3FD-0A87F3059420

urn:lsid:ipni.org:names:77215100-1

**Map 5.**
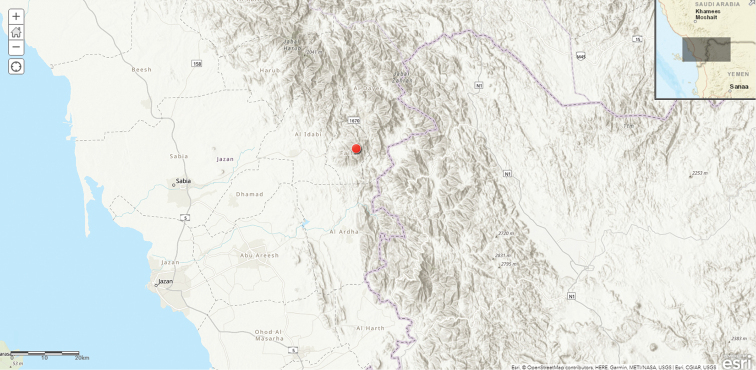
Distribution of Ceropegia lodarensis var. foetida.

**Figure 12.**
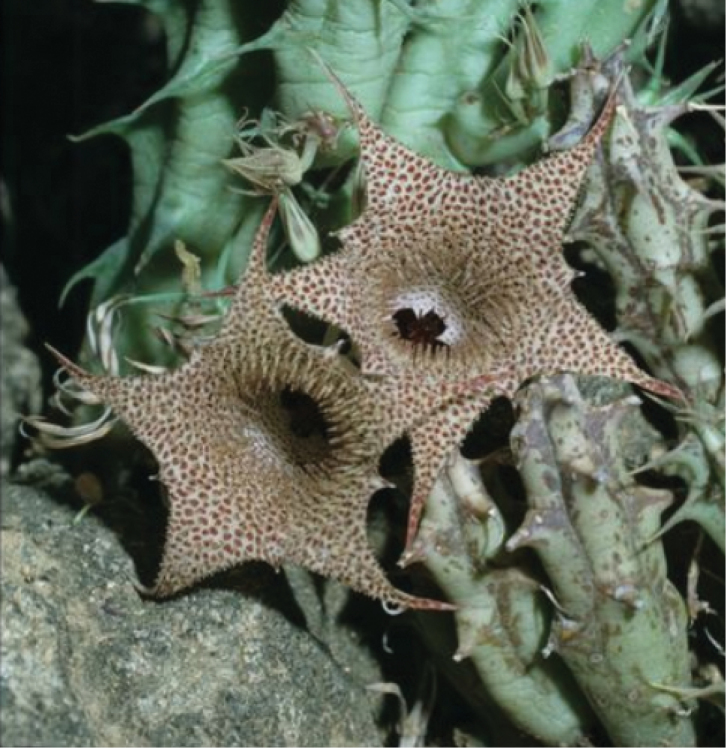
Ceropegia lodarensis var. foetida (*H. foetida*) Jabal Fayfa, Type. Reproduced from Plowes (2012).

**Figure 13.**
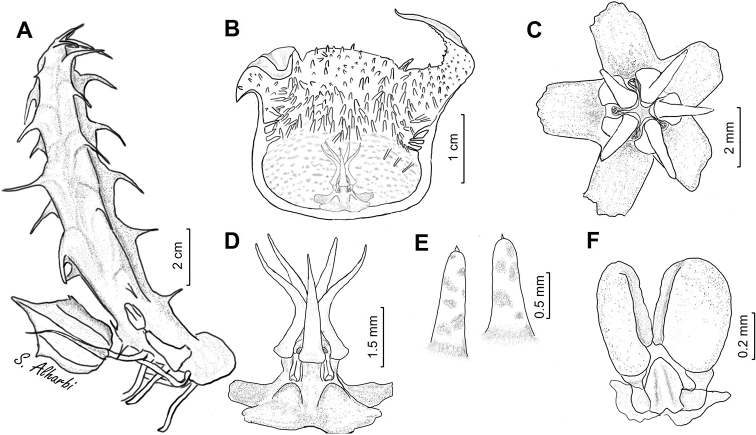
Ceropegia lodarensis var. foetida**A** branch **B** side view of dissected flower **C** face view of gynostegium **D** side view of gynostegium **E** papillae inside corolla in mouth of tube **F** pollinarium. Drawn from *Collenette 3743*, Jabal Fayfa.

Figs 12 , 13 ; Map 5

≡ Huernia foetida Plowes, Asklepios 114: 9 (2012).

#### Type.

Saudi Arabia – Jazan • *I.S. Collenette 3743* (holotype: K! [fl in spirit: 38892.000]); Jabal Fayfa, 80 km NE Jazan; 17°14.5296'N, 43°4.9368'E; alt. 1550 m; 31 Jul 1982.

#### Description.

***Branches*** up to 100 mm long; tubercles 7 mm long (including leaf-rudiment), 1.5 mm broad at base. ***Inflorescence*** bearing up to 4 flowers developing in gradual succession, flowers with very foetid odour; ***pedicel*** spreading and holding flower facing horizontally, tapering towards the point of flower attachment. ***Corolla*** 40 mm diam., campanulate; outside smooth, cream; inside cream with maroon dots changing to concentric short dashes in lower half of tube covered, except in lower third of tube with compressed conical papillae densely crowded and reach maximum size around mouth of tube (up to 1 mm long and 0.5 mm broad at base); ***tube*** 11 mm long, 10 mm broad at mouth, pentagonal; ***lobes*** spreading, deltoid, attenuate with deep groove at tip, ***intermediate lobes*** 1 mm long. ***Outer corona lobes*** 8 mm diam., five discrete rectangular lobes; ***inner lobes*** 3 mm long, 1 mm at base.

#### Distribution in Saudi Arabia.

Rare, known so far from Jabal Fayfa, 80 km NE Jazan, SW of the country (Plowes 2012).

#### General distribution.

Probably endemic to SW Arabian Peninsula known so far from Saudi Arabia.

#### Habitat and ecology.

Occurs in granitic outcrops at 1550 m alt (Collenette 1999).

#### Diagnosis.

Clearly distinct by its campanulate corolla that is dotted with maroon inside and has a very foetid odour when it opens.

#### Etymology.

Foetidus (Latin) smelly, for the strong, unpleasant smell of flowers.

#### Preliminary conservation status.

Ceropegia lodarensis var. foetida is estimated to have an EOO of 80.173 km^2^ (which would place the species in Critically Endangered, CR) and AOO of 88 km^2^ (which would place it in EN). The size of its populations and current threats are not well-known, but populations in mountainous areas in Saudi Arabia are likely impacted by agriculture, overgrazing, development and tourism. Therefore, var. foetida should be considered Data Deficient (DD).

### 4.3 Ceropegia lodarensis (Lavranos) Bruyns var. rubrosticta

Taxon classificationPlantaeGentianalesApocynaceae

(Plowes) Alharbi & Al-Qthanin comb. nov.

A9ACEB72-B99B-55E9-AC51-8B4BD8393CD6

urn:lsid:ipni.org:names:77215101-1

**Map 6.**
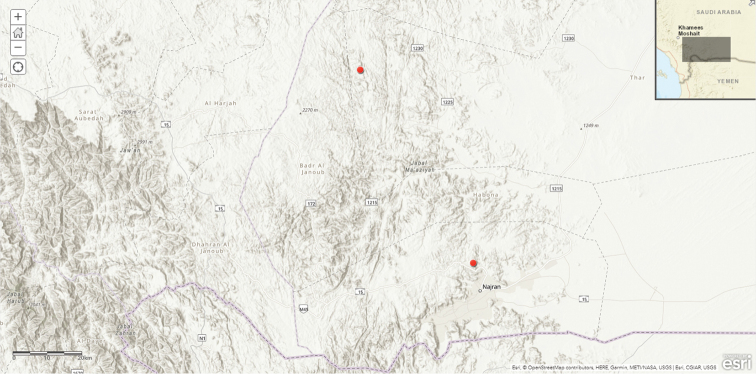
Distribution of Ceropegia lodarensis var. rubrosticta.

**Figure 14.**
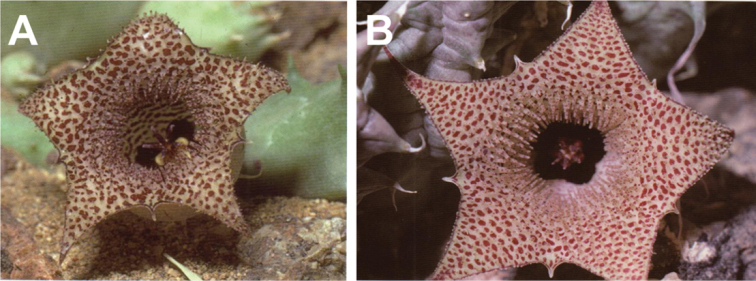
Ceropegia lodarensis var. rubrosticta (*H. rubrosticta*) **A** ex *S Collenette* s.n. sub *DP7639*, Al Jawshan, 70 km NW Najran (Type) **B***Collenette 1482*, Jabal Manfah, 24 km NE Najran. Reproduced from Plowes (2012).

**Figure 15.**
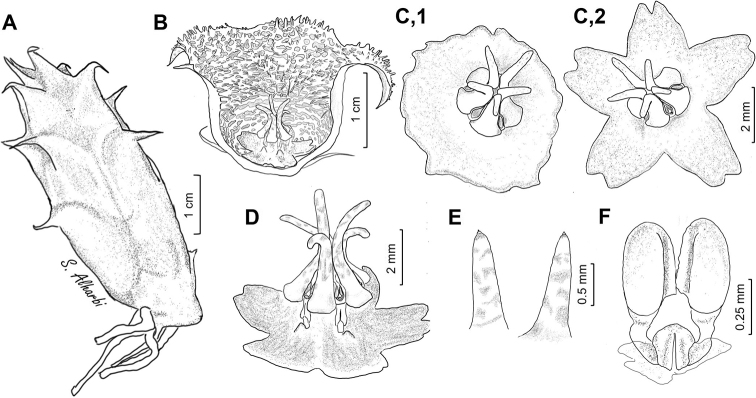
Ceropegia lodarensis var. rubrosticta**A** branch **B** side view of dissected flower **C** face view of gynostegium **D** side view of gynostegium **E** papillae inside corolla in mouth of tube **F** pollinarium. Drawn from (**A, B2, C**) *DP7639*, Al Jawshan, 70 km NW Najran; (**B1, D, E**) *Collenette 6059*.

Figs 14 , 15 ; Map 6

≡ Huernia rubrosticta Plowes, Asklepios 114: 11 (2012).

#### Type.

Saudi Arabia – Najran • *I.S. Collenette 1482* (holotype: k! herb. material); Jabal Manfah, 24 km NE Najran; 17°36.9386'N, 44°12.3742'E; alt. 1700 m; 30 Apr 1979.

#### Description.

***Branches*** 30–60 mm long, stout; tubercles 5.5–6 mm long (including leaf-rudiment), 2–3 mm broad at base. ***Inflorescence*** bearing 6 flowers developing in gradual succession from short peduncle, flowers with faint unpleasant smell; ***pedicel*** 11 mm long, 2 mm thick, ascending holding flower facing upwards, tapering sometimes towards the point of flower attachment; ***sepals*** 9.5 mm long, 2 mm broad at base, attenuate. ***Corolla*** 32 mm diam., campanulate; outside smooth, cream-speckled with pale maroon spots on the upper half of corolla tube; inside cream with rounded maroon spots or dashes changing to concentric broken lines in lower half of tube; ***papillae*** up to 1.5 mm long and 0.75 mm broad at base; ***tube*** 10 mm long, 12 mm broad at mouth, pentagonal; ***lobes*** 8 mm long, 7.5 mm broad at base, spreading with recurved apex, deltoid, acute concave at tip, ***intermediate lobes*** 1.5 mm long. ***Outer corona lobes*** 5 mm diam. fused into disc or a slightly disc-like with short subquadrate crenate; ***inner lobes*** 3 mm long, 1 mm at base.

#### Distribution in Saudi Arabia.

Rare, known only from Najran Region, SW Saudi Arabia (Plowes 2012).

#### General distribution.

Probably endemic to SW Arabian Peninsula, known so far only from Saudi Arabia

#### Habitat and ecology.

Concentrated amongst rounded granitic boulders at 500–1700 m alt. (Collenette 1999). Flowering: March-April.

#### Diagnosis.

The variety is most similar to the Ethiopian endemic *Huernia boleana*, from which it can be separated with flowers by the much more campanulate corolla that is wider than the long, pentagonal tube, the more conical papillae and the shorter inner coronal lobes (ca. 3 mm compared to ca. 6 mm in *H. boleana*) and with habit and habitat that is erect to decumbent amongst granitic rocks compared to *H. boleana* that is erect, pendulous or prostrate in basalt or sandstone.

It can be easily distinguished from the other varieties of *C.lodarensis* proposed here by the stout branches, flowers with more evenly-rounded spots inside that are more red in colour and by the more slender papillae.

#### Preliminary conservation status.

Ceropegia lodarensis var. rubrosticta has an estimated EOO of 97.188 km^2^ (which would place the species in CR) and AOO of 20 km^2^ (which would place it in EN). The size of populations and current threats are little known. Therefore, C. lodarensis var. rubrosticta should be considered Data Deficient (DD).

#### Additional specimens examined.

Saudi Arabia – Najran • *I.S. Collenette 6059* (K [fl in spirit: 51184.000]); Al Jawshan, 70 km NW Najran; 18°8.4287'N, 43°51.2486'E; alt. 1520 m.; 07 Mar 1987.

## Supplementary Material

XML Treatment for Ceropegia sect. Huernia

XML Treatment for Ceropegia macrocarpa

XML Treatment for Ceropegia khalidbinsultanii

XML Treatment for Ceropegia laevis

XML Treatment for Ceropegia lodarensis

XML Treatment for Ceropegia lodarensis (Lavranos) Bruyns var. lodarensis

XML Treatment for Ceropegia lodarensis (Lavranos) Bruyns var. foetida

XML Treatment for Ceropegia lodarensis (Lavranos) Bruyns var. rubrosticta

## Supplementary materials

Supplementary material 1 Appendices 1–3 Data type statistical data Explanation note Illustration shows how the plant parts were measured, Screen plot eigenvalues for identification of principal components and boxplot showing differences in morphological characters of *Huernia* species. https://binary.pensoft.net/file/516849This dataset is made available under the Open Database License (http://opendatacommons.org/licenses/odbl/1.0/). The Open Database License (ODbL) is a license agreement intended to allow users to freely share, modify, and use this Dataset while maintaining this same freedom for others, provided that the original source and author(s) are credited.Samah A. Alharbi, Rahmah N. Al-Qthanin
