# Supplementary material for: Taxonomic revision of Ceropegia sect. Huernia (Asclepiadoideae, Apocynaceae) in Saudi Arabia with three new combinations
Source: PhytoKeys. 2021 Mar 5;174:47–80. doi: 10.3897/phytokeys.174.58867 (PMC7954780; doi:10.3897/phytokeys.174.58867)
Supplement: Supplementary material 1 — Appendices 1–3 [file phytokeys-174-047-s001.docx]

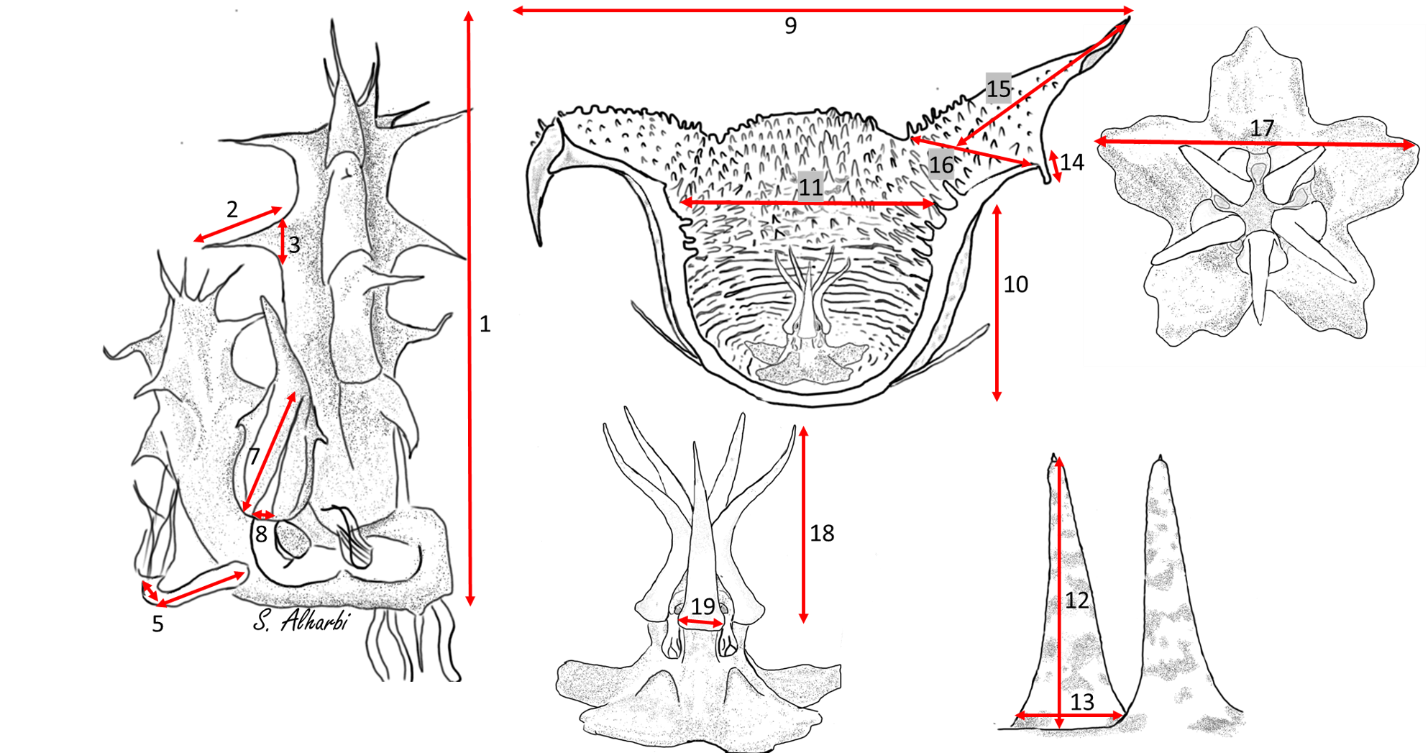


Appendix. 1 Illustration shows how the plant parts were measured. The numbers were related to the quantitative morphological characteristics shown in Table 1.


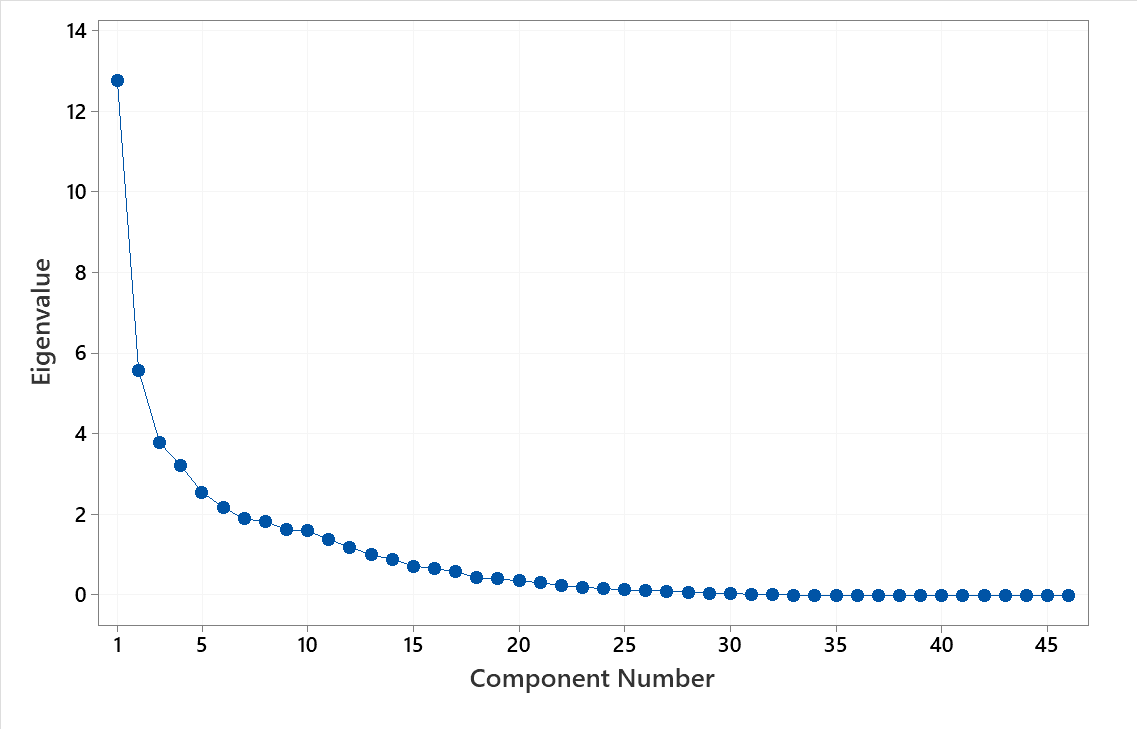


Appendix. 2 Screen plot Eigenvalues for identification of principal components.


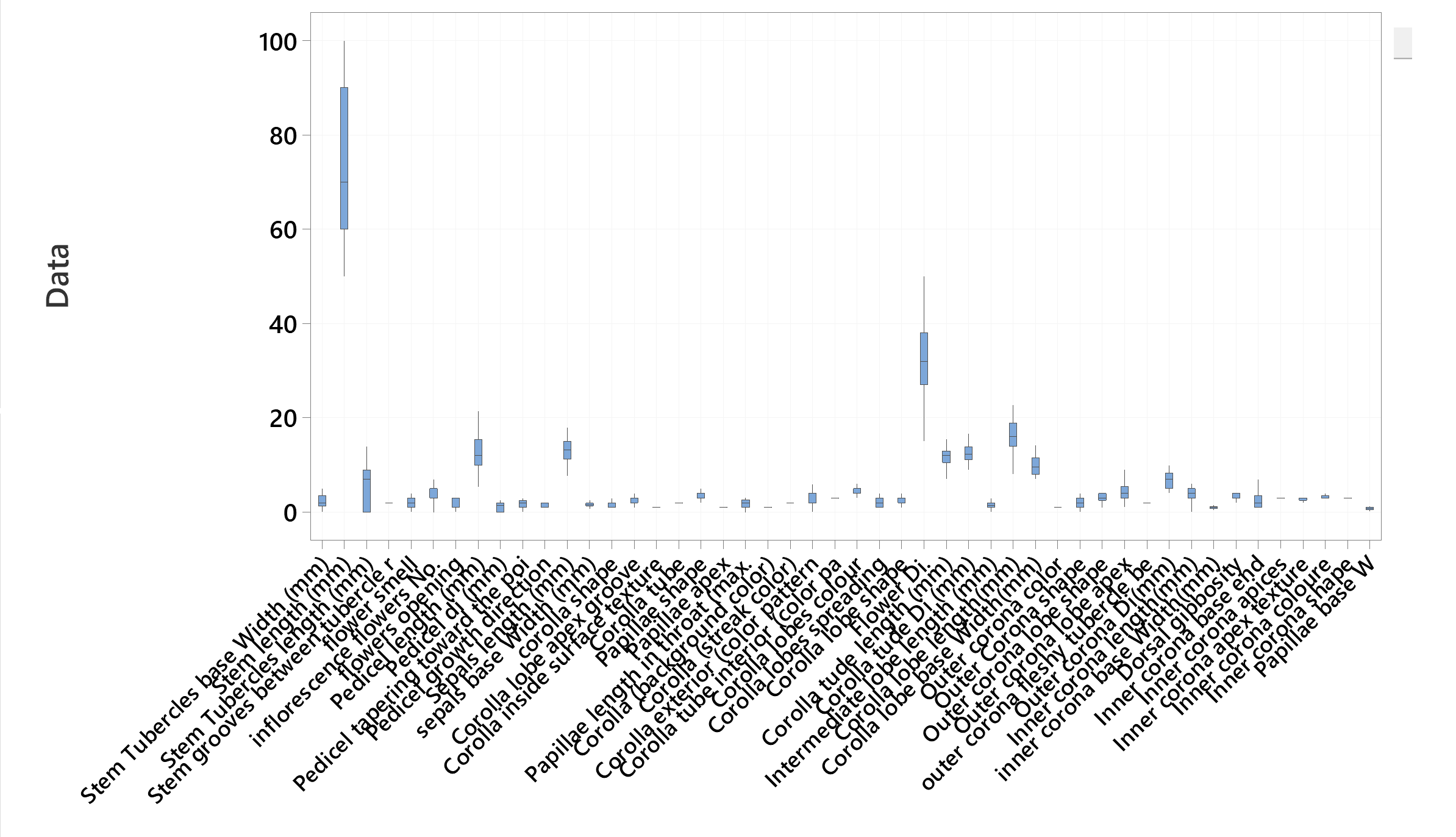


Appendix. 3 Boxplot showing differences in morphological characters of *Huernia* species.
